# Genetically Engineered Frameshifted YopN-TyeA Chimeras Influence Type III Secretion System Function in *Yersinia pseudotuberculosis*

**DOI:** 10.1371/journal.pone.0077767

**Published:** 2013-10-03

**Authors:** Ayad A. A. Amer, Tiago R. D. Costa, Salah I. Farag, Ummehan Avican, Åke Forsberg, Matthew S. Francis

**Affiliations:** 1 Department of Molecular Biology, Umeå University, Umeå, Sweden; 2 Umeå Centre for Microbial Research (UCMR), Umeå University, Umeå, Sweden; 3 Laboratory for Molecular Infection Medicine Sweden (MIMS), Umeå University, Umeå, Sweden; University of Helsinki, Finland

## Abstract

Type III secretion is a tightly controlled virulence mechanism utilized by many gram negative bacteria to colonize their eukaryotic hosts. To infect their host, human pathogenic *Yersinia* spp. translocate protein toxins into the host cell cytosol through a preassembled Ysc-Yop type III secretion device. Several of the Ysc-Yop components are known for their roles in controlling substrate secretion and translocation. Particularly important in this role is the YopN and TyeA heterodimer. In this study, we confirm that *Y. pseudotuberculosis* naturally produce a 42 kDa YopN-TyeA hybrid protein as a result of a +1 frame shift near the 3 prime of *yopN* mRNA, as has been previously reported for the closely related *Y. pestis*. To assess the biological role of this YopN-TyeA hybrid in T3SS by *Y. pseudotuberculosis*, we used *in cis* site-directed mutagenesis to engineer bacteria to either produce predominately the YopN-TyeA hybrid by introducing +1 frame shifts to *yopN* after codon 278 or 287, or to produce only singular YopN and TyeA polypeptides by introducing *yopN* sequence from *Y. enterocolitica*, which is known not to produce the hybrid. Significantly, the engineered 42 kDa YopN-TyeA fusions were abundantly produced, stable, and were efficiently secreted by bacteria *in vitro*. Moreover, these bacteria could all maintain functionally competent needle structures and controlled Yops secretion *in vitro*. In the presence of host cells however, bacteria producing the most genetically altered hybrids (+1 frameshift after 278 codon) had diminished control of polarized Yop translocation. This corresponded to significant attenuation in competitive survival assays in orally infected mice, although not at all to the same extent as *Yersinia* lacking both YopN and TyeA proteins. Based on these studies with engineered polypeptides, most likely a naturally occurring YopN-TyeA hybrid protein has the potential to influence T3S control and activity when produced during *Yersinia*-host cell contact.

## Introduction

Invertebrate and vertebrate hosts are potentially subject to a myriad of bacterial infections. Scores of these infectious agents are Gram-negative bacterial pathogens that colonize their eukaryotic hosts through a virulence strategy that involves having a type III secretion system (T3SS) as the centrepiece [1,2]. Similar systems also function in the biosynthesis of the flagellum motility organelle and in establishing mutualistic interactions between bacteria and their eukaryotic hosts. At least in pathogenic bacteria, target cell contact triggers a pre-assembled needle-like T3SS consisting of ~25 proteins spanning the bacterial envelope to become competent for delivery of newly synthesized effector toxins direct from the bacterial interior into the host cell cytosol in a one- or two-step process that presumably involves effector transit through a translocon pore formed in the host cell membrane [3]. At least three types of protein substrates are known to be secreted by a T3SS [4]; early substrates are those that contribute to the final phase of polymerizing the external needle appendage, middle substrates are pore-forming translocator proteins that bridge the gap between the protruding needle and host cell surface, thereby facilitating the passage of late substrates into the host cell interior. These late substrates are the effector toxins that harbour diverse enzymatic activities to manipulate host-cell signalization. This can affect many aspects of cell and host physiology – for instance immune system responsiveness, to promote bacterial survival in the host and host-to-host transmission [5].

This functional demarcation of substrate classes implies that their production and subsequent secretion is needed only at discrete phases during T3S activity. To ensure this concise temporal and spatial control, multiple layers of regulatory control are needed [1,6-10]. Common to all T3SSs appears to be a substrate switching mechanism which, following assembly of the needle extension, triggers a change in substrate secretion from early needle components to the middle translocators and late effectors. This notion is based on a plethora of studies that have dissected aspects of the complex crosstalk between YscU-like, YscP-like and YscI-like protein families that are highly conserved in both flagella and non-flagella T3SSs [11-24].

**Figure 1:**
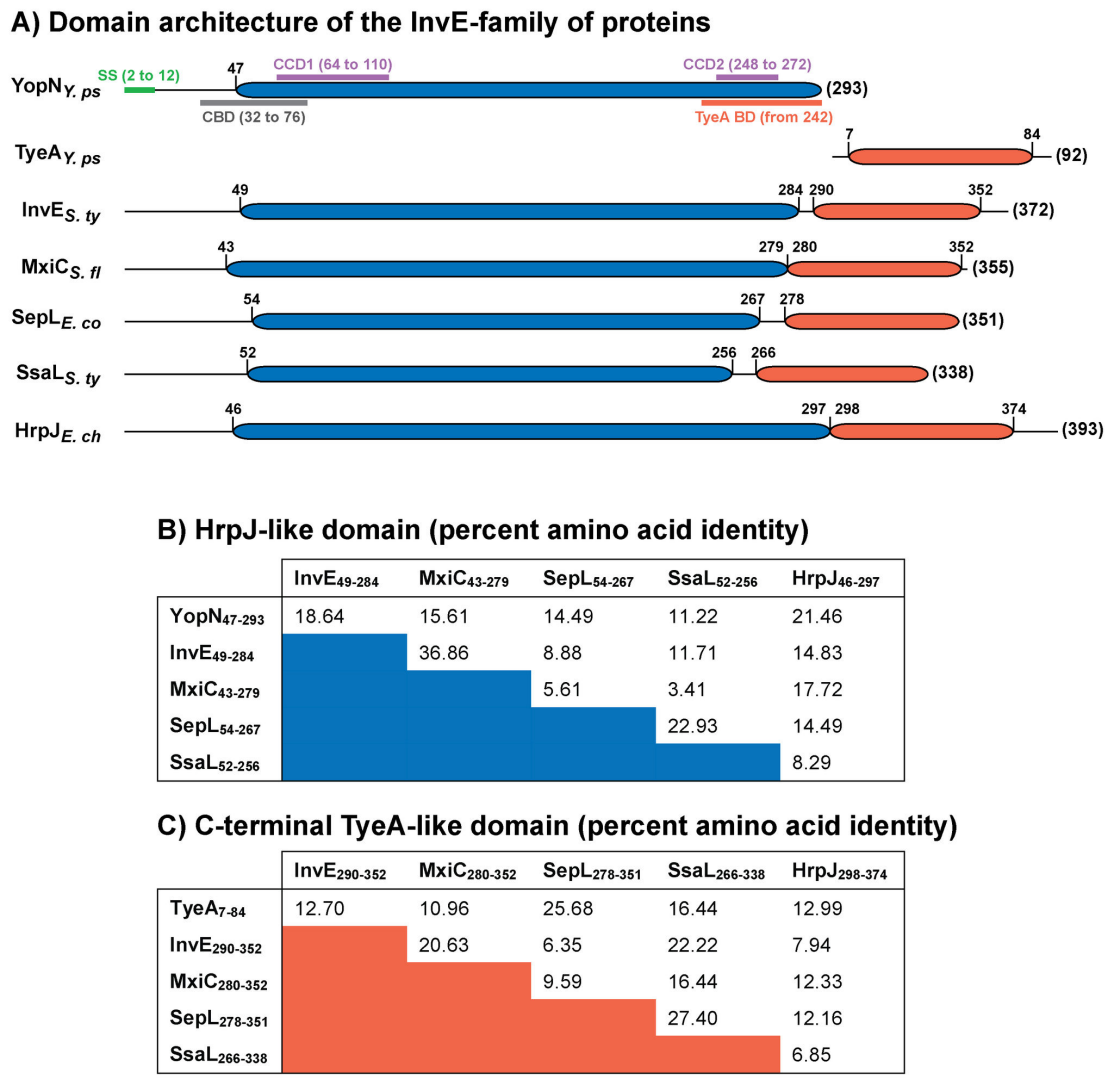
Domain architecture and sequence identity among the InvE-family of T3SS proteins. YopN and TyeA from human pathogen *Yersinia* sp. are two distinct polypeptides (A). In several other T3SSs, homologues to both YopN and TyeA exist as a single polypeptide (for example, InvE, MxiC, SepL, SsaL and HrpJ). Numbers in parentheses indicate the full length (in amino acids) of each protein. Other numbers indicate the bordering amino acids that demarcate YopN homology (blue shade) that is defined Pfam as a HrpJ-like domain (pfam07201), TyeA homology (orange shade) or functionally relevant regions of YopN (various coloured solid lines). The schematic illustration of YopN and TyeA homology domains within the InvE-family was derived from comprehensive multiple sequence alignments coupled to a Conserved Domain Database (CDD) [32,33]. SS, secretion signal [80]; CBD, T3S chaperone (YscB-SycN heterodimer) binding domain [92]; CCD1 and CCD2, coiled-coil domain 1 and 2 [61]; TyeA BD, TyeA binding domain [61,92]. Percent amino acid sequence identity between the InvE family of proteins was determined by BLASTP analysis for the N-terminal HrpJ-like domain (equivalent to YopN) (B) and the C-terminal TyeA-like domain (C). Representative sequences were retrieved from the NCIB genome database archived with the following GI reference numbers shown in parentheses: *Y*. *ps, Yersinia pseudotuberculosis* YopN (48634); *Y*. *ps* TyeA (48635); *S*. *ty, Salmonella enterica Typhimurium* InvE (16766203); *S*. *fl, Shigella flexneri* MxiC (12329090); *E*. *co, Escherichia coli* SepL (215267040); *S*. *ty* SsaL (16419933); *E*. *ch, Erwinia chrysanthemi* HrpJ (28628125).

It is also anticipated that a secretion order may exist among the middle and late secretion substrates. This is based on the assertion that a translocon pore should form in the host cell plasma membrane prior to the secretion of the translocated toxins. Indeed, accumulating genetic studies are providing evidence that in some bacteria middle substrates are prioritized for secretion over late substrates. A growing heterogeneous family of proteins headlined by InvE of *S. enterica Typhimurium* are being reported for their roles in ensuring translocator secretion before effector secretion in their respective bacteria. InvE directly recognizes translocator-chaperone complexes that may prioritize their secretion [25,26]. Alternatively, the C-terminus of SepL may specifically bind effector substrates to stall their T3S from enteropathogenic *Escherichia coli* [27-29] or MxiC may bind the system ATPase creating a blockade that similarly inhibits effector secretion by *Shigella flexneri* [19,30,31]. No matter how it is achieved, these studies identify intrinsic mechanisms for orchestrating hierarchical secretion among the T3S translocator and effector substrates. A Conserved Domain Database (CDD) [32] search revealed a distinct HrpJ-like domain (denoted pfam07201) architecture in all of them (Figure 1A), although only a modest amount of sequence identity is shared between them [33]. For example, amino acid identity within the HrpJ-like domain is highest (36.86%) between InvE and MxiC, but then sharply drops away for the others (Figure 1B).

InvE-family homologues were also reported within the plasmid encoded Ysc-Yop T3SS carried by the infamous *Yersinia pestis*, the etiological agent of plague, and the less aggressive foodborne enteropathogens *Y. enterocolitica* and *Y. pseudotuberculosis*. Intriguingly, this homology was partitioned over two proteins; YopN with a HrpJ-like domain displayed moderate identity to the N-terminus and TyeA followed with modest identity over the C-terminus of each InvE-family member (Figure 1A) [33]. The region of YopN containing the HrpJ-like domain was most identical at the amino acid level to HrpJ (21.46%) (Figure 1B), while TyeA amino acid sequence most closely resembled the C-terminal region of SepL (25.68%) (Figure 1C). The YopN and TyeA proteins do function as a 42kDa YopN-TyeA complex to control Yop substrate secretion [34-36]. Moreover, YopN function is required for the polarized translocation of T3S effectors into the host eukaryotic cell [35,37,38]. Curiously, *Y. pestis* but not *Y. enterocolitica* were observed to produce a singular 42 kDa YopN-TyeA hybrid polypeptide; a consequence of a +1 frame shift that occurs during translation of the 3’ -prime end of *yopN* mRNA. The produced hybrid protein was competent for general T3S control [39].

The mechanisms of Yop secretion control in *Yersinia* are complex and require input from multiple contributing proteins that function at different levels and in response to different environmental cues [9,10,24,40-44]. This study had the goal to further investigate the biological significance of the YopN-TyeA hybrid given the documented roles played by YopN and TyeA in Yop secretion control and their homology to the InvE-family. To do so, we first confirmed the natural production and T3S of the singular YopN-TyeA hybrid in *Y. pseudotuberculosis*. Next, an *in cis* site directed mutagenesis approach generated *Y. pseudotuberculosis* that either produced predominately the YopN-TyeA hybrid by introducing +1 frame shifts to *yopN* after codons 278 or 287, or produced only singular YopN and TyeA polypeptides by introducing *yopN* sequence from *Y. enterocolitica*. Like parental *Yersinia*, mutants that produced solely the YopN-TyeA hybrid maintained T3SS assembly and function *in vitro* and could also successfully establish systemic colonization during competitive infections of mice. In light of this functionality, a possible mechanism for regulating the natural formation of the YopN-TyeA hybrid was explored.

## Materials and Methods

### Bacterial Strains, Plasmids and Growth Conditions

Strains and plasmids used in this study are listed in Table 1. Routine bacterial culturing of *E. coli* and *Y. pseudotuberculosis* was performed at 37°C and 26°C respectively, typically in Luria Bertani (LB) broth. When examining protein expression and secretion from *Yersinia*, strains were grown in brain heart infusion (BHI) broth, both in minus calcium (BHI supplemented with 5 mM EGTA and 20mM MgCl_2_ – T3S permissive medium) and in plus calcium (2.5mM CaCl_2_ – T3S non-permissive medium) conditions. In both cases, bacteria were grown in the presence of 0.025% (v/v) Triton X-100. This treatment detached Yops prone to associate to the bacterial surface, thereby ensuring that our T3S analysis would include all Yops secreted beyond the bacterial envelope [45]. When appropriate, antibiotics at the following concentrations were used to select for plasmid maintenance during culturing: Carbinicillin (Cb) 100µg/ml, Chloramphenicol (Cm) 25µg/ml, and Kanamycin (Km) 50µg/ml.

**Table 1:** Strains and plasmids used in this study.

**Strains and plasmids**	**Relevant genotype or phenotype**	**Source or reference**
Strain		
*E. coli*		
DH5	F^−^, recA1, endA1, hsdR17, supE44, *thi*-1, gyrA96, relA1	Vicky Shingler
S17-1λ*pir*	*recA*, *thi*, *pro*, hsdR^-^ *M* ^+^, Sm^R^, <RP4:2-Tc:Mu:Ku:Tn7>Tp^R^	[86]
*Y. pseudotuberculosis*		
YPIII/pIB102	*yadA*::Tn5, Km^R^ (wild type)	[47]
YPIII/pIB75	pIB102, *yscU* in frame deletion of codons 25-329, Km^R^	[87]
YPIII/pIB75-26	pIB102, *yscU* and *lcrQ* double mutant, Km^R^	[45]
YPIII/pIB202	pIB102, *yscF* in frame deletion of codons 11-69, Km^R^	[88]
YPIII/pIB619	pIB102, *yopB* and *yopD* full length deletion, Km^R^	[89]
YPIII/pIB82	pIB102, near full length deletion of *yopN*, Km^R^	[90]
YPIII/pIB801a	pIB102, *tyeA* in frame deletion of codons 19-59, Km^R^	This study
YPIII/pIB8201a	pIB102, in frame double deletion of *yopN* and *tyeA*, Km^R^	This study
YPIII/pIB8214	pIB102, *yopN* allele with a missense mutation at codon 286 (Lys_AAA_→Ile_ATA_) to give a YopN_*Yps→Yen*_, Km^R^	This study
YPIII/pIB8205	pIB102, *yopN* allele with a +1 frameshift deletion mutation (‘T’) after codon 278 to give a YopN_278(F+1_)TyeA chimera, Km^R^	This study
YPIII/pIB8206	pIB102, *yopN* allele with a +1 frameshift deletion mutation (‘T’) after codon 278 and the conservative mutations at codons 283 and 284 (Gln_CAG→CAA_ and Arg_AGG→CGT_) that partially disrupts the presumed *tyeA* Shine-Dalgarno sequence to give a YopN_278(F+1_)_, SD_TyeA chimera, Km^R^	This study
YPIII/pIB8210	pIB102, *yopN* allele with a +1 frameshift deletion mutation (‘A’) after codon 287 to give a YopN_287(F+1_)TyeA chimera, Km^R^	This study
YPIII/pIB8211	pIB102, *yopN* allele with a +1 frameshift deletion mutation (‘A’) after codon 287 and the conservative mutations at codons 283, 284 and 285 (Ser_TCA→TCC_, Glu_GAG→GAA_ and Gly_GGT→GGC_) that partially disrupts the presumed *tyeA* Shine-Dalgarno sequence to give a YopN_287(F+1_)_, SD_TyeA chimera, Km^R^	This study
YPIII170/pIB102	*In cis* polar mutation of YPK_3687 in the parental background, Cm^R^, Km^R^	This study
YPIII170/pIB8201a	*In cis* polar mutation of YPK_3687 in the *yopN* and *tyeA* background, Cm^R^, Km^R^	This study
YPIII170/pIB8214	*In cis* polar mutation of YPK_3687 in the YopN_*Yps→Yen*_-producing background, Cm^R^, Km^R^	This study
YPIII170/pIB8205	*In cis* polar mutation of YPK_3687 in the YopN_278(F+1_)TyeA-producing background, Cm^R^, Km^R^	This study
YPIII170/pIB8206	*In cis* polar mutation of YPK_3687 in the YopN_278(F+1_)_, SD_TyeA-producing background, Cm^R^, Km^R^	This study
YPIII170/pIB8210	*In cis* polar mutation of YPK_3687 in the YopN_287(F+1_)TyeA-producing background, Cm^R^, Km^R^	This study
YPIII170/pIB8211	*In cis* polar mutation of YPK_3687 in the YopN_287(F+1_)_, SD_TyeA-producing background, Cm^R^, Km^R^	This study
YPIII/pIB8215	pIB102, *yopN* allele with a conservative mutation at codon 278 (Phe_TTT_→Phe_TTC_) to give a YopN_F278F_, Km^R^	This study
YPIII/pIB8216	pIB102, *yopN* allele with a missense mutation at codon 279 (Trp_TGG_→Phe_TTC_) to give a YopN_W279F_, Km^R^	This study
YPIII/pIB8217	pIB102, *yopN* allele with a deletion of codon 278 to give a YopN_Δ278F_, Km^R^	This study
YPIII/pIB8218	pIB102, *yopN* allele with a deletion of codon 279 to give a YopN_Δ279W_, Km^R^	This study
*Y. enterocolitica*		
8081/pYVe8081	clinical isolate, biotype 1b (serotype 0:8)	[91]
Plasmid		
pTZ57R/T	PCR cloning and sequencing vector, Cb^R^	Thermo Scientific
pMMB208	Expression vector, Cm^R^	[49]
pAA269	pMMB208 with full-length *yopN* and *tyeA* including native upstream SD sequences, Cm^R^	This study
pAA271	pMMB208 with chimeric *yopN*_278(F+1_)_, SD_ *tyeA* including native upstream SD sequences, Cm^R^	This study
pAA304	pMMB208 with full-length *yopN* and *tyeA-flag*® including native upstream SD sequences, Cm^R^	This study
pAA305	pMMB208 with full-length *yopN*_Yps→*Yen*_ and *tyeA-flag*® including native upstream SD sequences, Cm^R^	This study
pAA306	pMMB208 with chimeric *yopN*_278(F+1_)*tyeA-flag*® including native upstream SD sequences, Cm^R^	This study
pAA307	pMMB208 with chimeric *yopN*_278(F+1_)_, SD_ *tyeA-flag*® including native upstream SD sequences, Cm^R^	This study
pAA308	pMMB208 with chimeric *yopN*_287(F+1_)*tyeA-flag*® including native upstream SD sequences, Cm^R^	This study
pAA309	pMMB208 with chimeric *yopN*_287(F+1_)_, SD_ *tyeA-flag*® including native upstream SD sequences, Cm^R^	This study
pUA066	pNQ705-derived mutagenesis vector for the construction of a polar insertion in YPK_3687, Cm^R^	This study
pDM4	Suicide vector with oriR6K, *sacB*, Cm^R^	[46]
pAA256	*Sal*I/*Xba*I PCR fragment of *tyeA* with a in frame deletion of codons 19-59 in pDM4, Cm^R^	This study
pSF019	*Sal*I/*Xba*I PCR fragment flanking upstream of *yopN* and downstream of *tyeA* in pDM4, Cm^R^	This study
pAA251	*Sal*I/*Xba*I PCR fragment of *yopN* with a missense mutation at codon 286 (Lys_AAA_→Ile_ATA_) in pDM4, Cm^R^	This study
pAA242	*Sal*I/*Xba*I PCR fragment of *yopN* with a +1 frameshift deletion mutation (‘T’) after codon 278 in pDM4, Cm^R^	This study
pAA243	*Sal*I/*Xba*I PCR fragment of *yopN* with a +1 frameshift deletion mutation (‘T’) after codon 278 and the conservative mutations at codons 283 and 284 (Gln_CAG→CAA_ and Arg_AGG→CGT_) in pDM4, Cm^R^	This study
pAA247	*Sal*I/*Xba*I PCR fragment of *yopN* with a +1 frameshift deletion mutation (‘A’) after codon 287 in pDM4, Cm^R^	This study
pAA248	*Sal*I/*Xba*I PCR fragment of *yopN* with a +1 frameshift deletion mutation (‘T’) after codon 278 and the conservative mutations at codons 283, 284 and 285 (Ser_TCA→TCC_, Glu_GAG→GAA_ and Gly_GGT→GGC_) in pDM4, Cm^R^	This study
pAA252	*Sal*I/*Xba*I PCR fragment of *yopN* with a conservative mutation at codon 278 (Phe_TTT→_Phe_TTC_) in pDM4, Cm^R^	This study
pAA253	*Sal*I/*Xba*I PCR fragment of *yopN* with a missense mutation at codon 279 (Trp_TGG_→Phe_TTC_) in pDM4, Cm^R^	This study
pAA254	*Sal*I/*Xba*I PCR fragment of *yopN* with a deletion of codon 278 in pDM4, Cm^R^	This study
pAA255	*Sal*I/*Xba*I PCR fragment of *yopN* with a deletion of codon 279 in pDM4, Cm^R^	This study

### Mutant Construction

The various mutated *yopN* alleles were created by the overlap PCR method using the various primer pairs listed in Table S1. PCR fragments were cloned directly into pTZ57R/T using the InsTAclone PCR cloning strategy (Thermo Scientific) and each mutation confirmed by sequence analysis (Eurofins MWG Operon, Ebersburg, Germany). Confirmed DNA fragments were then lifted into the pDM4 suicide mutagenesis vector [46] following *Sal*I-*Xba*I restriction. *E. coli* S17-1λ*pir* harbouring the different mutagenesis constructs were used as the donor strains in independent conjugations with *Y. pseudotuberculosis* parent (YPIII/pIB102) [47]. Appropriate allelic exchange events were monitored by Cm sensitivity and sucrose resistance. All mutants were confirmed by a combination of PCR and sequence analysis. Significantly, each variant was introduced *in cis* on the *Y. pseudotuberculosis* virulence plasmid to ensure expression occurred in the context of native regulatory elements.

To generate a polar mutation in the YPK_3687 locus of various *Y. pseudotuberculosis* YPIII derived strains, we used the pUA066 mutagenesis vector. The pUA066 construct is based on pNQ705 and was generated by digestion with *Sal*I/*Xba*I and then ligation of a DNA fragment that was PCR amplified with the primer pair combination of pFpNQ066 and pRpNQ066 (Table S1) using DNA template derived from a boiled lysate of *Y. pseudotuberculosis* IP32953. Conjugal transfer of pUA066 into *Yersinia* involved a mating with *E. coli* S17-1λ*pir* carrying the mutagenesis vector. Disruption of YPK_3687 occurred via a single homologous recombination cross-in of pUA066. Verification of the disruption utilised PCR and a series of primer combinations including a pair intended to amplify the entire YPK_3687 open reading frame and another combination designed to amplified the 5-prime end of the YPK_3687, including the upstream flanking region, and part of the integrated pUA066 vector.

### Analysis of *In Vitro* Yop Synthesis and Secretion

Analysis of Yop synthesis and secretion by *Y. pseudotuberculosis* followed the procedure as previously described [45]. Samples of culture suspensions were taken to represent the total protein fraction, whereas the cleared bacteria-free supernatant corresponds to the secreted Yops fraction. Primary rabbit polyclonal antibodies recognizing YopN, YopD, YopE and DnaK were all a gift of Hans Wolf-Watz (Umeå University, Sweden), while those recognizing TyeA were a gift of Gregory Plano (University of Miami, USA). Detection used anti-rabbit antiserum conjugated with horse radish peroxidase (GE Healthcare, Buckinghamshire, United Kingdom) and Thermo Scientific Pierce ECL 2 Western Blotting Substrate to detect individual protein bands by western blotting.

### Intracytoplasmic Stability Assay

Intrabacterial protein stability was assessed by the method of Feldman and colleagues using Cm as the *de novo* protein synthesis inhibitor [48]. Protein fractions were analyzed by SDS-PAGE and Western blot. Steady state accumulated YopN or YopN-TyeA hybrid was detected by treatment of the PVDF membrane with rabbit polyclonal YopN antiserum, in combination with horseradish peroxidase conjugated anti-rabbit antibodies (Amersham Biosciences) and a homemade luminol-based detection kit.

### Generation of Constructs for Ectopic Expression of YopN and TyeA

Lysates of *Yersinia* parent and mutant bacteria was used in PCR to amplify the overlapping *yopN* and tyeA alleles on a single DNA fragment using the primer pair combinations listed in Table S1. Fragments were digested with *Bam*HI and *Eco*RI prior to ligation with similarly digested pMMB208 [49]. Confirmed clones were stored in *E. coli* S17-1λ*pir*, which was also used as donor in conjugal matings to mobilise the expression constructs into the Δ*yopN, tyeA* double mutant (YPIII/pIB8201a).

### Low Calcium Growth Measurements

The ability of *Yersinia* to grow at 37°C under high- and low-Ca^2+^ conditions was performed by measuring absorbance at 600nm (A_600_) of bacterial cultures grown in liquid Thoroughly Modified Higuchi’s (TMH) medium (minus Ca^2+^) or TMH medium supplemented with 2.5 mM CaCl_2_ (plus Ca^2+^) [50]. Growth phenotypes were compared to parental *Y. pseudotuberculosis* (YPIII/pIB102), which is defined as calcium dependent (CD), since it is unable to grow in the absence of Ca^2+^ at 37°C, and *Yersinia* lacking the *yscU* and *lcrQ* alleles (YPIII/pIB75-26) which is termed temperature sensitive (TS) reflecting its inability to grow at 37°C [45].

### YscF Surface Localization and Chemical Crosslinking

Overnight cultures from *Yersinia* strains were grown with shaking at 26°C in 2 ml of BHI broth supplemented with 2.5mM CaCl_2_. Subsequently, 0.1 volumes of bacterial suspension were sub-cultured into 3 ml fresh media and incubated for 3 hour at 37°C. After each culture was standardized by A_600_, 1 ml volumes were harvested by centrifugation at 8000*g* for 5 min at 4°C. Each bacterial pellet was gently resuspended in 1 ml of cold 20 mM HEPES, 2.5 mM CaCl_2_ (pH 8). Bacterial surface proteins were cross-linked for 30 min at ambient temperature with the non-cleavable, membrane-impermeable, amine-reactive cross-linker Pierce bis(sulfosuccinimidyl)suberate (BS^3^) (Thermo Scientific) at a final concentration of 5 mM. Cross-linking reactions were quenched for 15 min by addition of Tris-HCl (pH 8.0) to a final concentration of 20 mM. Cell fractions were collected by centrifugation at 12200*g* for 5 min at 4°C. Bacterial pellets were then resuspended in 100 µl of 1x SDS-PAGE loading buffer (50mM Tris-HCl, pH 6.8, 2% SDS, 0.1% Bromophenol blue, 10% Glycerol, 5% β-Mercaptoethanol) and analyzed by 18% acrylamide SDS PAGE and immunoblotting with rabbit anti-YscF polyclonal antiserum (a gift from Hans Wolf-Watz) that underwent several rounds of immunoadsorption with purified YscF to enhance its monospecificity.

### Non-Polarized Secretion During Target Cell Contact

Cultivation and infection of HeLa cell monolayers was performed using our standard methods [51,52]. After 3 hours post-infection, 500 µl from the overlaying DMEM media was carefully collected, clarified by centrifugation for 10 min at 4 °C, and the bacterial-free supernatant representing the secreted protein fraction was added to 4x SDS-PAGE sample buffer (200mM Tris-HCl, pH 6.8, 8% SDS, 0.4% Bromophenol blue, 40% Glycerol, 20% β-Mercaptoethanol). To detect total protein levels, the infected HeLa cells were harvested directly into 125 µl of 4x SDS-PAGE loading buffer. Equivalent volumes of the total and soluble fractions were subjected to SDS-PAGE and western blotting. Comparable loading was confirmed by using mouse monoclonal antibodies specific for the eukaryotic protein β–actin (Clone AC-74, Sigma-Aldrich). Yop levels were detected using rabbit polyclonal anti-YopE and anti-YopD antisera. By comparing the amount of protein secreted into the extracellular media (soluble fraction) to the total synthesized protein induced upon bacteria-host cell contact (total whole cell lysates fraction), the proportion of YopE and YopD secreted into the media and thus the degree of non-polarized secretion can be estimated. The assay does not measure effector injection capacities, so the degree of polarized translocation of the YopE cytotoxin directly into the host cell cytosol remains unknown. Placebo controls utilized mock infections with bacteria in the absence of cell monolayers and cell monolayers in the absence of bacteria.

### Bacterial Viability in the Presence of Eukaryotic Cells

A modified method of Bartra and co-workers [53] as described in earlier studies [45,54,55] was used to establish bacterial viability in the presence of murine macrophage-like J774 cells. In essence, bacteria lacking a fully functional T3SS are more readily phagocytosed and are therefore more susceptible to the antimicrobial effects of J774 cells. This reduced viability was determined by performing colony forming unit (CFU) counts for relevant bacterial strains in infected eukaryotic cell lysates.

### Mouse Co-Infections and Competitive Index Measurements

Disruption by polar insertion of the gene encoding for a 349 amino acid inner membrane oligo-dipeptide/nickel ABC transporter permease (annotated as YPTB0523 in *Y. pseudotuberculosis* IP32953) has no measurable effect on *Yersinia* virulence in the mouse model neither in single strain infections nor in competitive infections with the isogenic wild-type strain (UA, unpublished). Therefore, this mutation was introduced into our mutants by a single cross-over of the pUA066 mutagenesis plasmid. As well as creating a polar mutation in the equivalent gene in *Y. pseudotuberculosis* YPIII (annotated as YPK_3687), integration of the mutagenesis plasmid conferred to these newly generated double mutants a Cm^R^ marker for counter-selection against Cm^S^ parental bacteria. Retention of the pIB102 virulence plasmid was verified with our standard *in vitro* Ysc-Yop synthesis and secretion assay. Comparable growth rates (monitored by A_600_) and corresponding CFU counts of all bacteria were also performed.

Female eight-week-old BALB/c mice (Taconic, Denmark) were given food and water *ad libitum*. Then groups of five mice were deprived of food and water 16 h prior to oral infection. For infection, bacteria were grown overnight in 50 ml LB broth at 26°C, then pelleted and serially diluted in sterile tap water supplemented with 150 mM NaCl. Serial dilutions were plated to record CFU/ml and their corresponding A_600_ measured to establish the volume of culture needed to inoculate 50 ml of sterile drinking water with 2.5 x 10^9^ viable mutant bacterial cells (Cm^R^) and 2.5 x 10^9^ viable parental bacterial cells (Cm^S^). Mice were allowed to drink from this inoculated water for 6 hours. Measurement of CFU was again performed to calculate the amount of Cm^R^ bacteria in the inoculation water, which was expressed as an input percentage of the total inoculated dose (Cm^S^ + Cm^R^). At day 4 post infection, spleens were harvested aseptically in sterile PBS, homogenized, and plated for bacterial CFU analysis to determine the amount of viable Cm^R^ bacteria, and this was expressed as an output percentage of the total recovered population. In turn, the competitive index was determined as the ratio of percent Cm^R^ output versus percent Cm^R^ input.

### Ethics Statement

The infection studies were performed in strict accordance with the Swedish Bioethical Guidelines for care and use of laboratory animals. The protocol was approved by The Umeå Committee on the Ethics of Animal Experiments (Permit Number: A-60-10).

## Results

### *Y. pseudotuberculosis* Naturally Produce and Secrete a YopN-TyeA Hybrid

**Figure 2:**
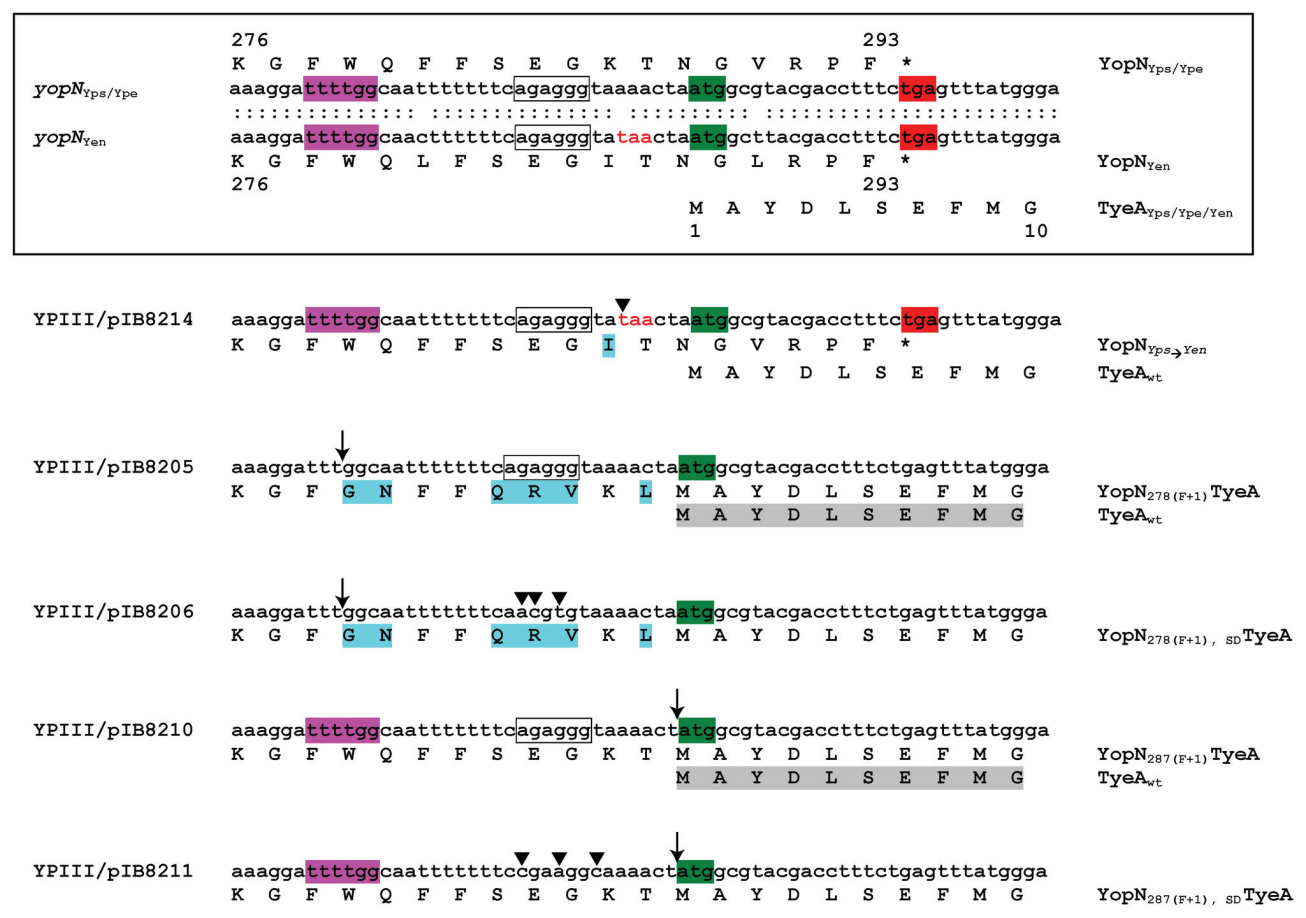
Region of sequence overlap between YopN and TyeA. Comparison of the nucleotide and amino acid sequence in YopN and TyeA derived from *Y. pseudtotuberculosis* (Yps), *Y. pestis* (Ype) and *Y. enterocolitica* (Yen) (boxed panel). Nucleotide sequence of the sense strand is given in lower case font with identity between *yopN*_Yps/Ype_ sequence and *yopN*_Yen_ sequence indicated by the colon symbol (:). Numbers indicated they amino acid sequence that is given in upper case font either above (for *yopN*_Yps/Ype_) or below (for *yopN*_Yen_) the gene sequence. The *yopN* termination codon is indicated by red highlight and the *tyeA* initiation codon in green highlight and the upstream putative Shine-Dalgarno sequence is boxed. The first 10 amino acid residues of TyeA are identical in all three *Yersinia* species. As described by others [39], the putative pausing site (‘ttttgg’) for instigating a +1 frame-shift to create a YopN-TyeA hybrid is presented in magenta highlight. The out-of-frame stop codon (‘taa’) just upstream of the *tyeA* start that would prevent hybrid formation via +1 frame-shifting in *Y*. *enterocolitica* is given in red font. Shown below the boxed panel are the mutations used to modulate YopN-TyeA hybrid formation in *Y*. *pseudotuberculosis*. The first mutation was a missense mutation (▼) at codon 286 (Lys_AAA_→Ile_ATA_) to introduce an out-of-frame ‘taa’ stop codon that abolished hybrid formation (YPIII/pIB8214; YopN_*Yps*→*Yen*_). The second mutation was a +1 frameshift deletion mutation (removal of ‘T’) after codon 278 (↓) to give a YopN_278_(_F+1_)TyeA chimera (YPIII/pIB8205). The third mutation was a +1 frameshift deletion mutation (‘T’) after codon 278 (↓) combined with conservative mutations (▼) at codons 283 and 284 (Gln_CAG→CAA_ and Arg_AGG→CGT_) that partially disrupts the presumed *tyeA* Shine-Dalgarno sequence to give a YopN_278_(_F+1_)_, SD_TyeA chimera (YPIII/pIB8206). The fourth mutation was a +1 frameshift deletion mutation (removal of ‘A’) after codon 287 (↓) to give a YopN_287_(_F+1_)TyeA chimera (YPIII/pIB8210). The fifth mutation was the same +1 frameshift deletion mutation (removal of an ‘A’) after codon 2878 (↓) combined with conservative mutations (▼) at codons 283, 284 and 285 (Ser_TCA→TCC_, Glu_GAG→GAA_ and Gly_GGT→GGC_) that partially disrupts the presumed *tyeA* Shine-Dalgarno sequence to give a YopN_287_(_F+1_)_, SD_TyeA chimera (YPIII/pIB8211). Altered amino acid sequence in YopN prior to the *tyeA* initiation codon is indicated in blue highlight. Gray highlight reflects the cessation of TyeA production as a singular polypeptide courtesy of disrupting its upstream Shine-Dalgarno sequence.

**Figure 3:**
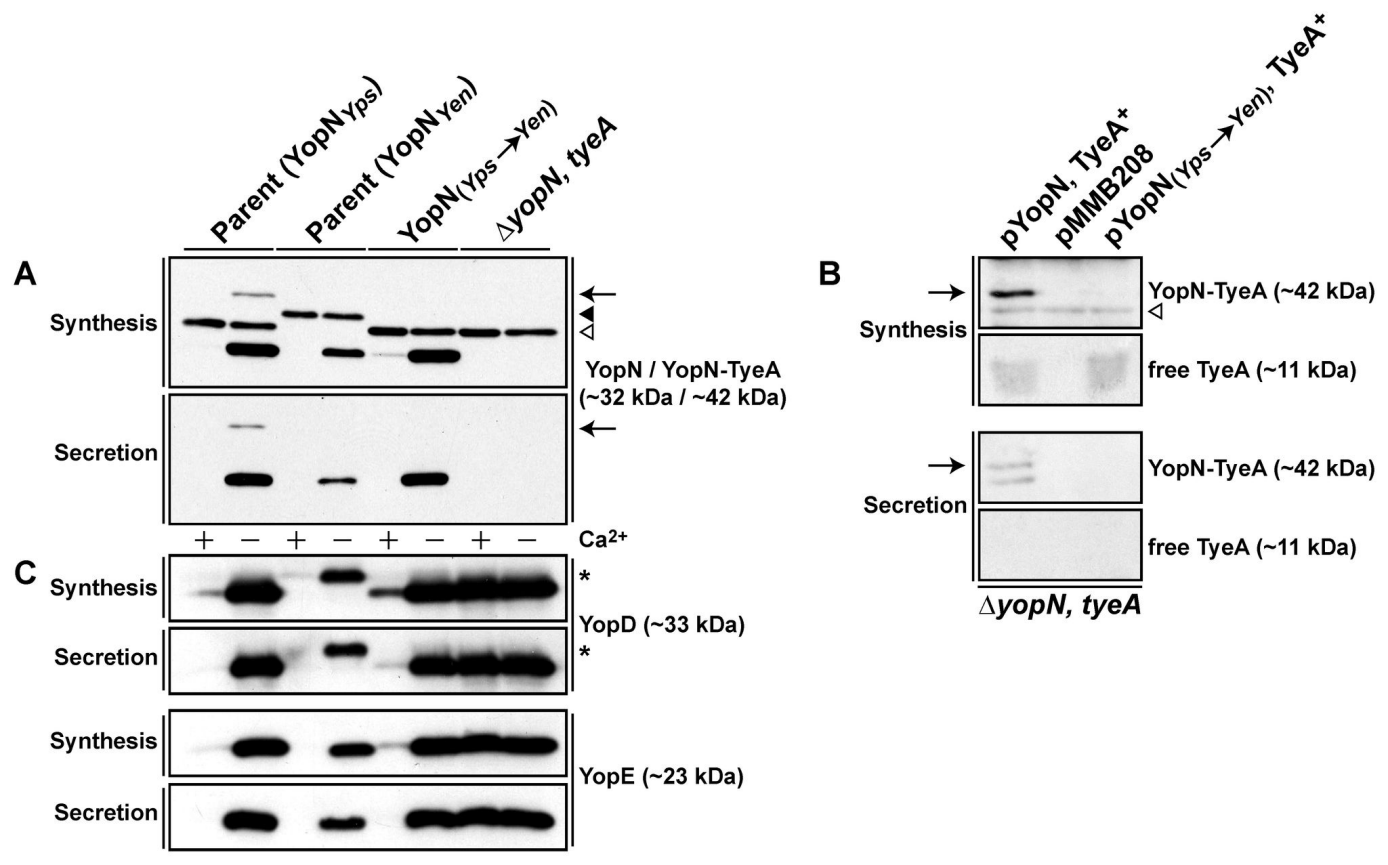
Analysis of naturally produced YopN-TyeA hybrid synthesis and secretion by *Y. pseudotuberculosis*. Overnight cultures of *Y. pseudotuberculosis* were sub-cultured into BHI medium in the presence (+) or absence (-) of calcium ions at 26°C for 1 hour and at 37°C for 3 hours. Protein in the total bacterial suspension (Synthesis) and free in the cleared culture supernatant (Secretion) were collected, fractionated by 12% acrylamide SDS-PAGE, wet-blotted onto PDVF membrane and then detected using rabbit polyclonal anti-YopN (A), anti-TyeA (B) and also anti-YopD and anti-YopE (C) antibodies. The arrow (←) is pointing toward the ~42 kDa YopN-TyeA hybrid. The open (∇) arrowhead identifies non-specific protein bands uniquely recognised by the anti-YopN and anti-TyeA antisera in protein samples derived from *Y. pseudotuberculosis*. The closed (▼) arrowhead indicates a non-specific protein band recognised by the anti-YopN antiserum in protein samples derived from *Y. enterocolitica*. The asterisk (*) highlights the altered mobility of the YopD product derived from *Y. enterocolitica*. In A and C, lanes are represented by: Parent (YopN_*Yps*_), *Y. pseudotuberculosis* YPIII/pIB102; Parent (YopN_*Yen*_), *Y. enterocolitica* 8081/pYVe8081; YopN_*Yps*→*Yen*_, *Y. pseudotuberculosis* YPIII/pIB8214; Δ*yopN, tyeA*, YPIII/pIB8201a. In B, lanes are *Y. pseudotuberculosis* Δ*yopN, tyeA* (YPIII/pIB8201a) also containing pYopN, TyeA^+^ (pAA304), empty vector (pMMB208) or pYopN_(Yps→*Yen*)_, TyeA^+^ (pAA305). Approximate molecular mass values shown in parentheses were deduced from primary amino acid sequences.

*Y. pestis* can produce and secrete a singular polypeptide consisting of a ~42 kDa hybrid of YopN and TyeA that was the result of a +1 frame shift during translation of the 3´-end of the *yopN* mRNA [39]. This hybrid was also a substrate of the Ysc-Yop T3SS. In contrast, a similar hybrid was not produced by *Y. enterocolitica* because any +1 frame-shift along the *yopN* mRNA would result in a premature stop codon immediately upstream of, and in the same reading frame as translated *tyeA* mRNA [39]. However, the *yopN* nucleotide sequences from *Y. pseudotuberculosis* and *Y. pestis* are identical (Figure 2). This would suggest that *Y. pseudotuberculosis* could also naturally produce a YopN-TyeA product. To examine for this, bacteria were grown in BHI broth restrictive (with Ca^2+^) or permissive (without Ca^2+^) for T3S to examine the *in vitro* synthesis and secretion profile of YopN. During growth in T3S permissive conditions, parental *Y. pseudotuberculosis* could produce and secrete a ~32 kDa protein that is YopN (Figure 3A). Interestingly, an additional Ca^2+^-regulated slower migrating band of ~42 kDa in both synthesis and secretion fractions was also recognized by the anti-YopN antisera; this band is consistent with the expected mass of a YopN-TyeA hybrid protein (Figure 3A). Critically, this band was not observed in synthesis and secretion fractions derived from an isogenic mutant of *Y. pseudotuberculosis* lacking both *yopN* and *tyeA* or from parental *Y. enterocolitica* (Figure 3A)*.*

In an effort to confirm natural YopN-TyeA chimeric production, initially we used anti-TyeA polyclonal antibodies to directly detect *in cis* production of native singular TyeA (~11 kDa) or native TyeA produced as a hybrid (~42 kDa). However, in our hands this was unsuccessful (data not shown), possibly due to low level production or a high rate of TyeA turnover. To circumvent this, we ectopically expressed the native *yopN* and *tyeA* alleles from an IPTG inducible promoter harboured on the pMMB208 expression plasmid (pAA304). Despite uncoupling regulatory control from the Ysc-Yop regulators, the gene synteny remained identical to that present on the virulence plasmid. From lysates derived from the Δ*yopN, tyeA* null mutant ectopically co-producing native YopN and TyeA, a ~42 kDa product in both synthesis and secreted fractions could be detected with anti-TyeA (Figure 3B). Additionally, the anti-TyeA antibodies also detected a diffuse band representing the free ~11 kDa TyeA product in the synthesis fraction only (Figure 3B).

**Figure 4:**
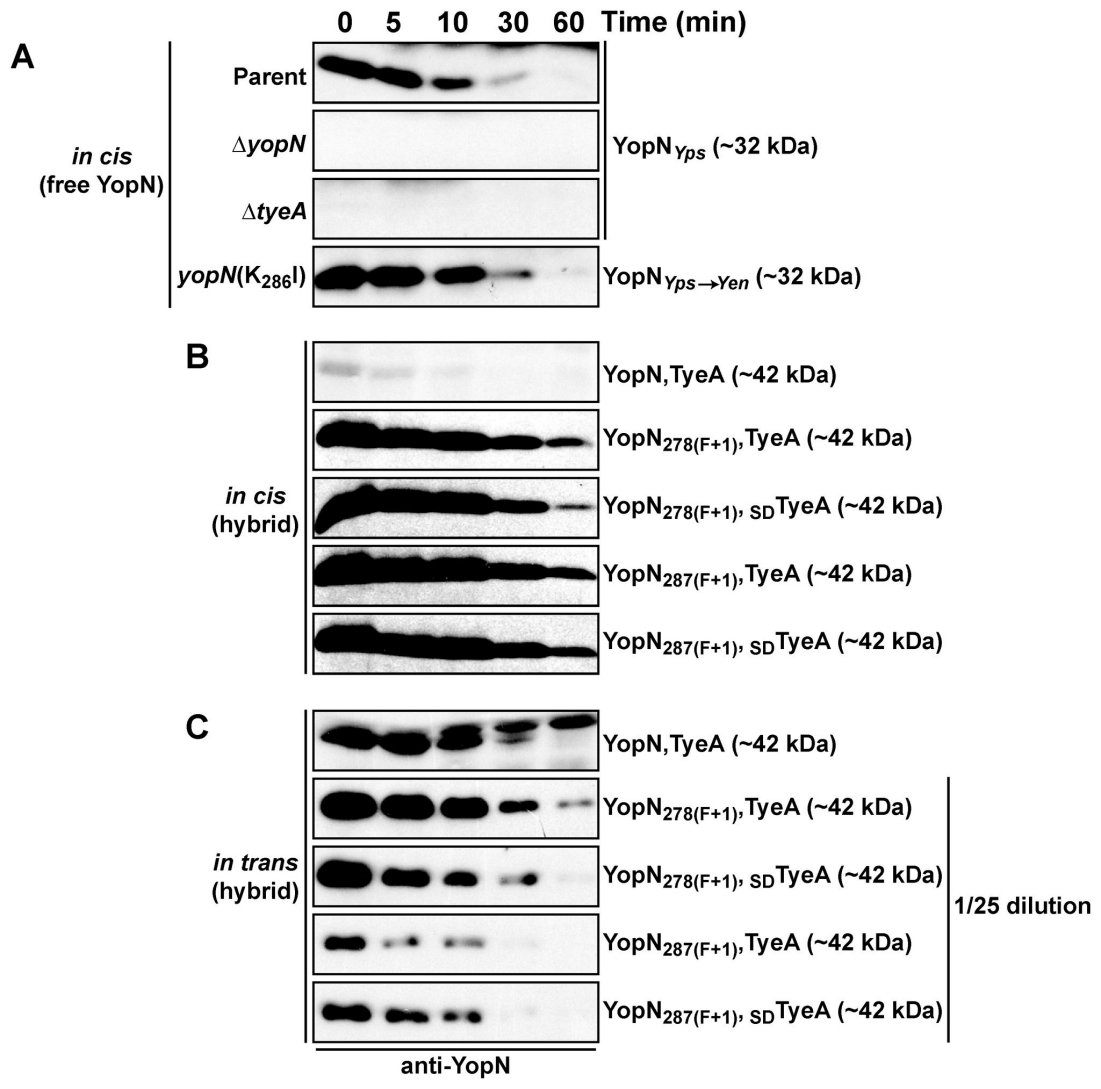
Intrabacterial stability of pre-formed pools of genetically engineered YopN-TyeA chimeras. Bacteria were first cultured for 1 hour in non-inducing (plus 2.5 mM CaCl_2_) BHI broth at 37°C either without (A and B) or with 0.4 mM IPTG (C). The protein synthesis inhibitor chloramphenicol (50 µg/ml) was added at time point 0 minutes (min). Samples were then collected at this and subsequent time points. Protein levels associated with pelleted bacteria were detected by Western blot using polyclonal anti-YopN antiserum to detect singular YopN produced *in* *cis* (A) or YopN produced as a hybrid with TyeA derived from *in* *cis* production (B) or IPTG inducible ectopic *in* *trans* production (C). Note that the majority of samples in C were diluted by a factor of 25 to reduce the amount of material subjected to gel fractionation. In A, samples are derived from: Parent (YopN_*Yps*_), YPIII/pIB102; Δ*yopN*, YPIII/pIB82; Δ*tyeA*, YPIII/pIB801a; YopN_*Yps→Yen*_ (YopN_K286I_), YPIII/pIB8214. In B, samples are derived from: Parent (YopN_*Yps*_), YPIII/pIB102; YopN _278(F+1)_TyeA, YPIII/pIB8205; YopN _278(F+1), SD_TyeA, YPIII/pIB8206; YopN _287(F+1)_TyeA, YPIII/pIB8210; YopN _287(F+1), SD_TyeA, YPIII/pIB8211. In C, samples are derived from *Y. pseudotuberculosis* Δ*yopN, tyeA* (YPIII/pIB8201a) also containing pYopN, TyeA^+^ (pAA304), pYopN_278(F+1)_, TyeA^+^ (pAA306), pYopN_278(F+1), SD_, TyeA^+^ (pAA307), pYopN_287(F+1)_, TyeA^+^ (pAA308), or pYopN_287(F+1), SD_, TyeA^+^ (pAA309). Approximate molecular mass values shown in parentheses were deduced from primary amino acid sequences.

To further confirm the contributions of both *yopN* and *tyeA* sequence in this hybrid, using site-directed mutagenesis the 3-prime *yopN* nucleotide sequence of *Y. pseudotuberculosis* was manipulated to generate the substitution K_286_I that resembled the *yopN* allele from *Y. enterocolitica*, which does not naturally produce the YopN-TyeA hybrid (Figure 2) [39]. The resulting mutant producing the YopN_*YpsYen*_ variant failed to produce or secrete a ~42 kDa product either when produce *in cis* (Figure 3A) or *in trans* when produced under the control of an IPTG inducible promoter harboured on the pMMB208 expression plasmid (pAA305) (Figure 3B). However, the free ~32 kDa product of singular YopN (Figure 3A) and ~11 kDa product of free TyeA (Figure 3B) were synthesized as normal. Interestingly, the inability to produce the ~42 kDa YopN-TyeA product in bacteria producing YopN_*YpsYen*_ did not negate the ability of these bacteria to maintain Ca^2+^-dependent control over the synthesis and secretion of middle and late Yop substrates, such as YopD and YopE respectively (Figure 3C). In contrast, complete removal of the *yopN* and/or *tyeA* alleles lead to the constitutive synthesis and secretion of YopD and YopE (Figure 3C and data not shown). Moreover, bacteria lacking *tyeA* could not maintain steady state levels of YopN (Figure 4A), suggesting that YopN stability and function depends on the presence of TyeA. We also confirmed that steady state levels of YopN_*YpsYen*_ were equivalent to native YopN (Figure 4A).

Taken together, these data are all consistent with the ability of *Y. pseudotuberculosis* to naturally produce a ~42 kDa YopN-TyeA singular polypeptide presumably as a result of a +1 frame shift during translation of the 3´-end of *yopN* mRNA. Moreover, this product undergoes Ca^2+^-regulated secretion via the Ysc-Yop T3SS. This corroborates occurrence of a similar sized product produced and secreted by the Ysc-Yop system in *Y. pestis* [39].

### Stable Production of Genetically Engineered YopN-TyeA Chimeras in *Y. pseudotuberculosis*

In prokaryotes (including viruses) and eukaryotes, programmed frame-shifting events are an important translational control mechanism for regulating the production of diverse functioning proteins [56-60]. The ~42 kDa hybrid protein naturally produced by *Y. pestis* and *Y. pseudotuberculosis* involved a frame-shifting event that fused the translation of *yopN* to overlapping *tyeA*, the products of which are essential mediators of T3S control. Although the levels of hybrid production and secretion are significantly lower than when produced as separate entities, we wondered if this hybrid is biologically relevant for T3S function in *Yersinia*. In order to investigate this, we utilized site directed mutagenesis to engineer *in cis* mutations in *yopN* that resulted in the artificial production of predominantly YopN-TyeA chimeras by *Y. pseudotuberculosis*. The first mutation was a +1 frame-shift directly introduced after *yopN* codon 278 by removal of a single ‘T’ nucleotide. This generated bacteria that produced a YopN-TyeA fusion – designated YopN _278(F+1_)TyeA – that consisted of native YopN amino acid until residue 278, followed by an altered sequence between residues 279 and 287, prior to the switch to TyeA specific coding sequence (Figure 2). This means that the extreme YopN C-terminus encompassing residues 288 to 293 are replaced by unadulterated N-terminal TyeA sequence. Similarly, a second strain was generated by introducing a +1 frame shift after *yopN* codon 287 by removal of an ‘A’ nucleotide located immediately upstream of the *tyeA* start codon. The result was a bacterium able to produce a YopN-TyeA fusion termed YopN _287(F+1_)TyeA, which incorporated native YopN sequence until residue 287, but was then followed by TyeA sequence. Once again, the extreme six residue YopN C-terminus was replaced by the beginning of TyeA (Figure 2). As these two mutants still left upstream of *tyeA* an uncharacterised but intact putative Shine Dalgarno (SD) sequence, albeit displaced by n−1 in the second mutant, they could conceivably still produce trace amounts of TyeA as a single (free) polypeptide entity. This was addressed by generating two additional mutants in which this putative SD sequence was conservatively ‘scrambled’ as much as possible without altering the *yopN* coding sequence. This resulted in two new mutants designated YopN _278(F+1_)_, SD_TyeA and YopN _287(F+1_)_, SD_TyeA respectively (Figure 2).

The stability of these four chimeras in the presence of endogenous proteases was examined. The larger ~42 kDa products synthesized *in cis* were easily detectable with anti-YopN antisera and remained as stable as the smaller ~32 kDa singular YopN polypeptide produced by parental *Y. pseudotuberculosis* (compare Figure 4B with Figure 4A). Additionally, all larger synthetic ~42 kDa variants accumulated in greater abundance, in contrast to the natural hybrid product that was barely detectable (Figure 4B). At this stage we have no firm grasp on why this might be the case. To determine whether the engineered YopN-TyeA(~42 kDa) variants displayed similar stability to the naturally formed hybrid produced by the parental strain, it was therefore necessary to establish a series of expression constructs that placed the various overlapping *yopN* and *tyeA* alleles PCR amplified from parent and mutant bacteria under an IPTG promoter on pMMB208. Ectopic *in trans* expression in the Δ*yopN, tyeA* double mutant now afforded sufficiently elevated production levels to detect stability of the natural hybrid (Figure 4C). Although it was necessary to load 25 times less protein material derived from the synthetic YopN-TyeA chimeric strains (i.e. diluted by a factor of 25) compared to the parental strain, their stability was essentially comparable to the native hybrid with the exception of YopN_287(F+1),_ TyeA that was a little less stable (Figure 4C).

Since free TyeA could be functional and bias the behavior of individual synthetic YopN-TyeA hybrids, it was also necessary to explore its status in the constructed strains. Antibodies raised against TyeA recognized the *in cis* produced ~42 kDa band representing artificially produced chimeric YopN-TyeA hybrids, but not the ~11 kDa band of free TyeA from these mutants or from parental bacteria (data not shown). To circumvent this, the pMMB208-derived expression constructs described for the stability assays (see Fig 4C) were again used to measure TyeA synthesis and secretion. Using anti-TyeA antibodies, we could once more detect high levels of the ~42 kDa band when ectopically expressed in *Yersinia* lacking *yopN* and *tyeA* (Figure S1). In contrast, the ~11 kDa band of free TyeA was clearly detected only when co-expressing the native *yopN* and *tyeA* alleles in the synthesis fraction, with possibly very low level expression of free TyeA detectable from the two constructs expressing the hybrids YopN _287(F+1_)TyeA and YopN _287(F+1_)_, SD_TyeA (Figure S1). Thus, if any free TyeA is produced in the four engineered chimeric strains, it is so low as to be essentially undetectable by western blot and consequently would likely not interfere with the function of YopN that is produced as part of the YopN-TyeA hybrid.

Hence, it was evident from this series of experiments that we successfully genetically manipulated *Y. pseudotuberculosis* to specifically produce a range of stable YopN-TyeA chimeras suitable to investigate their functional relevance to *Yersinia* biology.

### Secreted YopN-TyeA Hybrids Maintain *In Vitro* Yops Secretion Control

The current working hypothesis suggests that a tetra-complex of YopN, together with the cognate T3S chaperones YscB and SycN, as well as TyeA act together as a secretion plug located at the cytoplasmic face of the inner membrane to prevent entry of Yop substrates into the secretion channel [34-36,38]. When the T3S apparatus is competent for secretion, environmental cues such as target cell contact or calcium depletion are anticipated to alter conformation of the YscF needle in a way that permits secretion of YopN. Once the secretion plug is removed, the T3SS can engage with and secrete the raft of middle and late Yop substrates. Thus, to investigate the impact of YopN-TyeA chimera production on T3SS activity, we began by investigating the degree to which the YscF needle component was secreted and polymerized at the bacterial surface – the final step in the assembly of an active Ysc-Yop T3SS. In our assay, visualization of YscF polymerization was aided by the presence of the non-membrane permeable chemical crosslinker BS^3^. With the exception of the *yscF* null mutant used as an antibody specificity control, monomeric YscF that was located in the bacterial cytoplasm and protected from the membrane impermeable crosslinker was detected in all samples (Figure S2). Parental bacteria could also secrete YscF that was readily cross-linked by BS^3^ to form higher order structures indicative of the T3S needle (Figure S2). In contrast, surface-located YscF was completely absent in the T3SS-defective full-length *yscU, lcrQ* deletion mutant, even though cytoplasmic located monomeric YscF protected from the non-membrane permeable crosslinker was visualized (Figure S2). Critically, YopN-TyeA chimera production by bacteria did not impact on their ability to produce higher order YscF structures at the bacterial surface (Figure S2). Hence, chimeric-produce bacteria assemble the Ysc-Yop T3SS that is competent for secretion of early substrates such as the YscF needle component.

**Figure 5:**
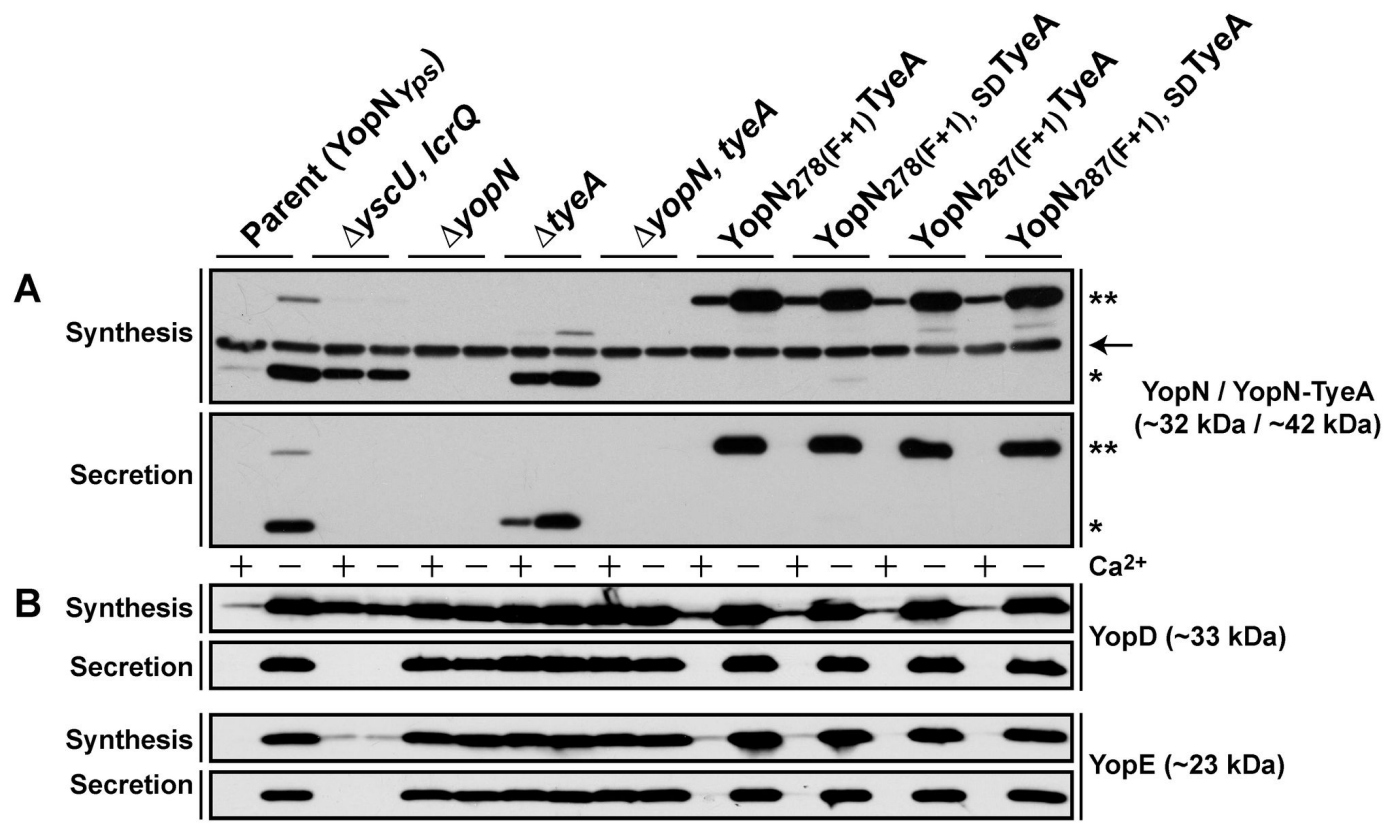
Analysis of YopN-TyeA hybrid synthesis and secretion. Overnight cultures of *Y. pseudotuberculosis* were sub-cultured into BHI medium in the presence (+) or absence (-) of calcium ions at 26°C for 1 hour and at 37°C for 3 hours. Protein in the total bacterial suspension (Synthesis) and free in the cleared culture supernatant (Secretion) were collected, fractionated by 12% acrylamide SDS-PAGE, wet-blotted onto PDVF membrane and then detected using rabbit polyclonal anti-YopN (A) and also anti-YopD and anti-YopE (B) antibodies. The arrow (→) point towards a non-specific protein band recognised by the anti-YopN antiserum. The single asterisk (*) highlights the single YopN polypeptide, while the double asterisk (**) indicates the larger YopN-TyeA hybrid protein. Lanes: Parent (YopN_*Yps*_), YPIII/pIB102; Δ*yscU, lcrQ* double mutant, YPIII/pIB75-26; Δ*yopN* null mutant, YPIII/pIB82; Δ*tyeA* null mutant, YPIII/pIB801a; Δ*yopN, tyeA* double mutant, YPIII/pIB8201a; YopN _278(F+1)_TyeA, YPIII/pIB8205; YopN _278(F+1), SD_TyeA, YPIII/pIB8206; YopN _287(F+1)_TyeA, YPIII/pIB8210; YopN _287(F+1), SD_TyeA, YPIII/pIB8211. Approximate molecular mass values shown in parentheses were deduced from primary amino acid sequences.

Next we examined if the YopN-TyeA chimeras could be secreted by the assembled T3SS during bacterial growth in BHI broth restrictive (plus Ca^2+^) and permissive (minus Ca^2+^) for T3S. Having already confirmed by western blot the presence of both YopN and TyeA sequence in the synthetic hybrids, for convenience we used only anti-YopN antisera in subsequent western blot analyses of their synthesis and secretion profiles. Parental bacteria produced and secreted both YopN alone (~32 kDa) and a YopN-TyeA hybrid (~42 kDa) (Figure 5A). Once again, it was evident that the engineered ~42 kDa YopN-TyeA hybrids accumulated to greater levels than did the smaller ~32 kDa singular YopN polypeptide (Figure 5A). As noted earlier [34,61,62], a Δ*tyeA* null mutant has lost control of T3S activity, producing and secreting YopN during growth in both low and high calcium media (Figure 5A). Interestingly, the Δ*tyeA* null mutant also produced a smaller YopN-TyeA_20-59_ hybrid product, consistent with the reduced size of truncated and inactivated TyeA (Figure 5A). Secretion was T3SS-dependent because a strain devoid of the YscU – an integral inner membrane component of the Ysc-Yop T3SS – failed to secrete YopN. Interestingly, YopN-TyeA hybrid producing bacteria did not cause any deviation in the synthesis and secretion profiles of the so-called middle (e.g. YopD) and late (e.g. YopE) Yop substrates, since they were all comparable to parental bacteria (Figure 5B). On the other hand, the single Δ*yopN* and Δ*tyeA* mutants along with the double Δ*yopN, tyeA* mutant had all lost general control with Yop substrate synthesis and secretion being constitutive regardless of the calcium concentration (Figure 5B). Thus, it appears that engineered YopN-TyeA hybrids all have the capacity to maintain tight control over Yop secretion reminiscent of when they are produced as two separate polypeptides [34-36]. This happens despite the higher steady-state accumulation of each individual hybrid. At this stage, we can only speculate that the reason for increased protein levels involves some aspect of translation efficiency and/or product stability not measurable by assays utilized in this study.

Deregulated defects in Yop secretion control correspond to aberrant growth patterns in low calcium at elevated temperature. Therefore, in parallel we measured growth of our *Yersinia* mutants in TMH growth medium (low calcium) and supplemented with 2.5 mM CaCl_2_ (high Ca^2+^) at 37 °C. Growth of parental bacteria followed a typical calcium-dependent profile, where growth was observed only in the presence of calcium (Figure S3). Significantly, this was similar to the growth profiles of all four YopN-TyeA hybrid producing bacteria (Figure S3), corroborating their intact Yops secretion control. In contrast, the single Δ*yopN* and Δ*tyeA* mutants along with the double Δ*yopN, tyeA* mutant that no longer had control over Yops synthesis and secretion, were all rendered completely temperature sensitive for growth regardless of a high or low Ca^2+^ concentration (Figure S3). Altogether, these data suggest that YopN-TyeA hybrids maintain *yop* regulatory control, at least during growth under these standard laboratory conditions.

### YopN-TyeA Hybrid Function in Effector Translocation

**Figure 6:**
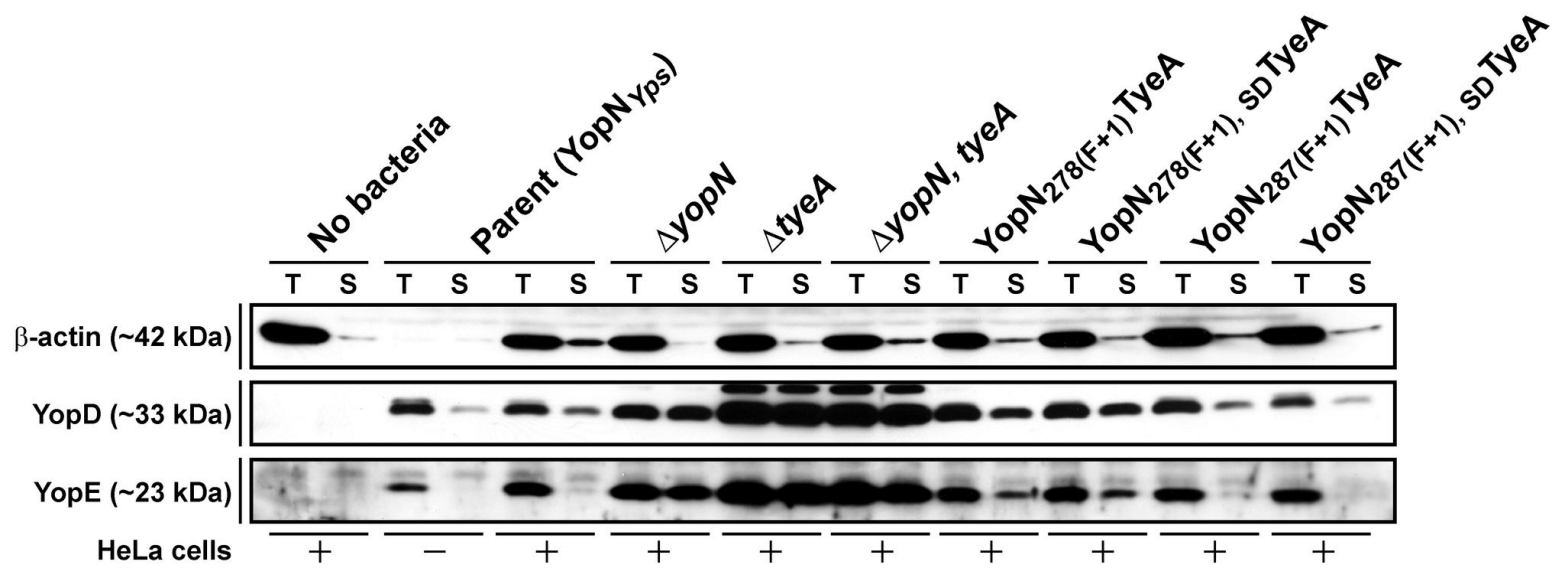
Polarized translocation of YopE by YopN-TyeA hybrid producing bacteria. HeLa cells were infected with parental and mutated *Y. pseudotuberculosis* strains. The cell-free culture supernatant (S) and total cellular material (T) was then analysed for YopE and YopD by ECL-Western blot using rabbit anti-YopE and anti-YopD serum. The extent of eukaryote cell cytosolic material in each fraction was indicated by a western blot probing for host derived β–actin. Lanes: No bacteria, Mock infection with HeLa cell monolayer alone: Parent (YopN_*Yps*_), YPIII/pIB102 either in the absence (−) or presence (+) of a HeLa cell monolayer; Δ*yopN* null mutant, YPIII/pIB82; Δ*tyeA* null mutant, YPIII/pIB801a; Δ*yopN, tyeA* double mutant, YPIII/pIB8201a; YopN _278(F+1)_TyeA, YPIII/pIB8205; YopN _278(F+1), SD_TyeA, YPIII/pIB8206; YopN _287(F+1)_TyeA, YPIII/pIB8210; YopN _287(F+1), SD_TyeA, YPIII/pIB8211. Approximate molecular mass values shown in parentheses were deduced from primary amino acid sequences.

Although recently challenged by a study proposing a two-step translocation model [63], Yop effector delivery into target eukaryotic cells has long been considered a one-step polarized mechanism that avoids wasteful effector substrate secretion into the extracellular environment [43,64,65]. In fact, *yopN* or *tyeA* mutant bacteria that have lost the ability to control Yop secretion *in vitro* also secrete Yops in a non-polarized fashion into the extracellular milieu when in contact with eukaryotic cells. As a result, subsequent *yopN* and *tyeA* mutant effector injection capacities are reduced [34,61,62,64,66]. Hence, the degree of non-polarized Yops secretion during host cell contact by *Y. pseudotuberculosis* producing hybrid YopN-TyeA polypeptides was measured. We compared two different fractions from infected HeLa cell monolayers; the first was the clarified extracellular supernatant (non-polarized secreted protein fraction) and the second was whole cell lysates (total protein fraction associated with bacteria, HeLa cells and in the supernatant). Very little Yops were detected in the supernatant fraction of HeLa cell infections with parental *Y. pseudotuberculosis*, despite high levels of protein available in the total protein pool (Figure 6). This observation reflects the central tenet that Yops are directly delivered into cells and are seldom released free into the environment. This contrasts with the *yopN* and/or *tyeA* deletion mutants that liberate far greater amounts of Yop material free into the extracellular environment (Figure 6), which is indicative of their reduced effector injection capacities as described previously [34,61,62,64,66]. For reasons currently unknown, *Y. pseudotuberculosis* lacking *tyeA* display greater de-repression than does the single *yopN* mutant. For bacteria producing engineered YopN _278(F+1_)TyeA and YopN _278(F+1_)_, SD_TyeA hybrid polypeptides, their capacity for Yops translocation was inferior as evidenced by the slight elevation in non-polarized Yops secretion into the extracellular environment during infection of tissue culture cell monolayers (Figure 6). In contrast, bacteria producing either YopN _287(F+1_)TyeA or YopN _287(F+1_)_, SD_TyeA still maintained polarized Yops secretion suggesting that these bacteria deliver Yops into HeLa cells with efficiencies reminiscent of parental bacteria (Figure 6). Hence, all four hybrid-producing bacteria maintain far superior control over T3SS activity than do bacteria lacking *yopN* and/or *tyeA*. The reduction observed for YopN _278(F+1_)TyeA and YopN _278(F+1_)_, SD_TyeA hybrid-producing bacteria is consistent with these variants producing a YopN module having the most altered C-terminal sequence (i.e. after codon 278; see Figure 2). Critically, this fault in target cell contact stimulated T3S control is not evident when examining low Ca^2+^-dependent induction *in vitro* in standard laboratory growth medium (see Figure 5).

**Figure 7:**
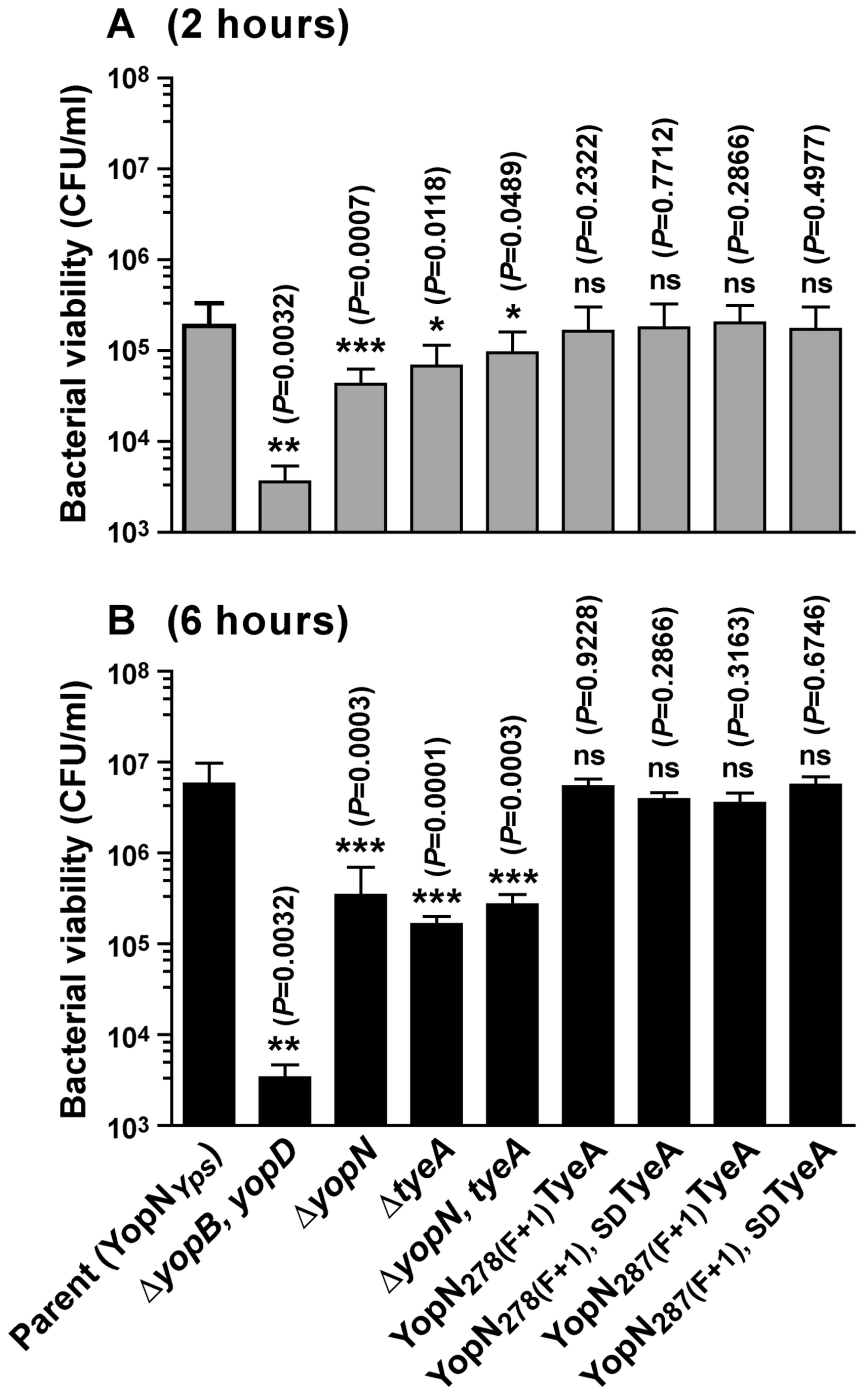
Formation of YopN-TyeA hybrids does not compromise *in vitro* T3SS activity. *Y. pseudotuberculosis* strains were used to infect murine macrophage-like J774-1 cells. Bacterial cells with a compromised T3SS were more rapidly phagocytosed and killed by these immune cells. Bacterial viability as measured by CFU/ml was determined at 2 hours (A) and 6 hours (B) post-infection and is expressed as a mean of 4 independent assays ± the standard deviation. Strains: Parent (YopN_*Yps*_), YPIII/pIB102; Δ*yopB, yopD* double mutant, YPIII/pIB619; Δ*yopN* null mutant, YPIII/pIB82; Δ*tyeA* null mutant, YPIII/pIB801a; Δ*yopN, tyeA* double mutant, YPIII/pIB8201a; YopN _278(F+1)_TyeA, YPIII/pIB8205; YopN _278(F+1), SD_TyeA, YPIII/pIB8206; YopN _287(F+1)_TyeA, YPIII/pIB8210; YopN _287(F+1), SD_TyeA, YPIII/pIB8211. Data sets were analyzed using the non-parametric two-tailed Mann-Whitney *U*-test. Analysis was performed using GraphPad Prism version 5.00 for Windows. Differences between mutants and parent (*yopN*_wt_) with a p-values < 0.05 were considered significant (*, ** and ***). ns – not statistically different.

In parallel, we measured the capacity of our YopN-TyeA hybrid producing bacteria to resist phagocytosis and killing by J774A.1 macrophage-like immune cells [45,53-55], which is a hallmark of Ysc-Yop T3S activity [67]. In principal, any bacteria with a compromised T3SS will be phagocytosed by immune cells, exposing these internalized bacteria to potent and effective anti-microbial killing strategies. In contrast, an active T3SS will protect bacteria from phagocytosis so they can proliferate extracellularly. Bacterial infections were observed up to 6h post-infection. At 2h and 6h post-infection, the viability of bacteria associated with host cells was determined by measuring colony forming units (CFU). Importantly, the translocation defective and growth restricted Δ*yopB, yopD* null mutant cannot resist immune cell phagocytosis and is efficiently killed, which dramatically restricts the recovery of viable bacteria at 2h (Figure 7A, P *=*0.0032, **) and again at 6h post-infection (Figure 7B, P *=*0.0032, **). While not to the same extent as the Δ*yopB, yopD* null mutant, removal of *yopN* and/or *tyeA* is also a serious impediment to sustaining bacterial viability in the face of immune cell activity at both early (Figure 7A, P *<*0.05, * and ***) and late time points (Figure 7B, P *<*0.005, ***), corroborating severe defects in polarized secretion of effector Yops (see Figure 6) [34,61,62,64,66]. On the other hand, all four YopN-TyeA hybrid producing bacteria efficiently resisted phagocytosis and killing by J774A.1 macrophage-like immune cells at both early and late time-points to a similar degree as parental bacteria (Figure 7, *P*>0.05, no significant difference). This suggests that the deficiencies in polarized secretion observed for YopN _278(F+1_)TyeA and YopN _278(F+1_)_, SD_TyeA producing bacteria does not impact negatively on their resistance to immune cell engulfment and killing. When considered altogether, these *in vitro*-based assays suggest that the YopN-TyeA hybrids can support T3SS function.

### Virulence Attenuation of *Yersinia* Producing YopN-TyeA Hybrids

**Figure 8:**
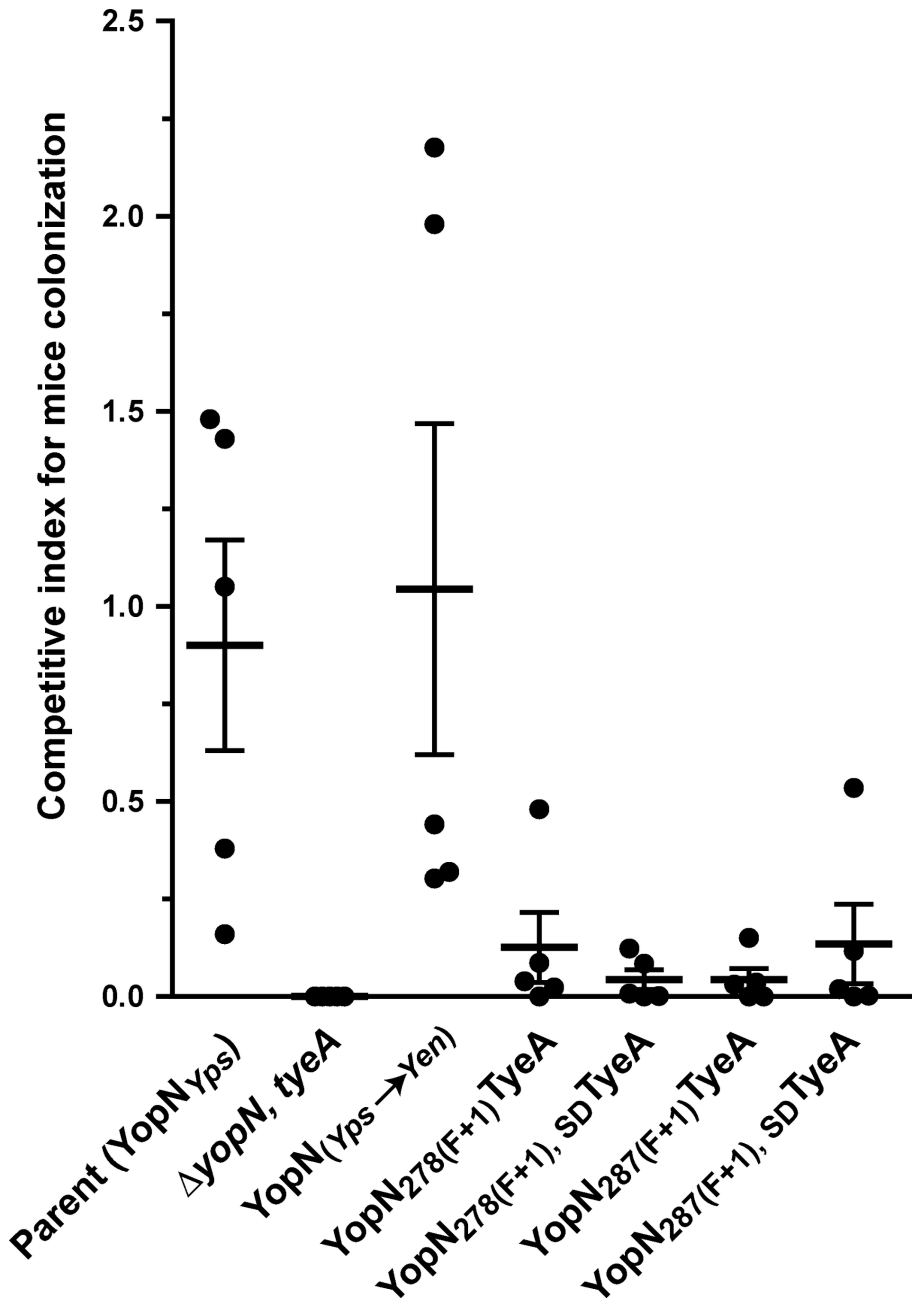
Competitive index for mice colonization. *Y. pseudotuberculosis* mutants with defective *yopN* alleles as well as parental bacteria (*yopN* ^+^) were manipulated to confer resistance to chloramphenicol by virtue of introducing a polar mutation into the YPK_3687 allele. These strains were used together with parental bacteria (Cml^S^) to co-infect groups of five mice via intentional contamination of their drinking water. Bacteria recovered from extracted spleens were measured by CFU/ml after four days of infection. The competitive indices (CI) were determined according to the footnotes in Table S2. Each symbol (•) reflects the CI derived from an individual mouse and the horizontal line is the mean of five mice ± the standard error. Strains: Parent (YopN_*Yps*_), YPIII170/pIB102; YopN_*Yps*→*Yen*_, *Y. pseudotuberculosis* YPIII170/pIB8214; Δ*yopN, tyeA* double mutant, YPIII170/pIB8201a; YopN _278(F+1)_TyeA, YPIII170/pIB8205; YopN _278(F+1), SD_TyeA, YPIII170/pIB8206; YopN _287(F+1)_TyeA, YPIII170/pIB8210; YopN _287(F+1), SD_TyeA, YPIII170/pIB8211. Note that all strains harbour a polar insertion in YPK_3687 (i.e. strain designation ‘YPIII170’).

If the YopN-TyeA hybrid can fully support Ysc-Yop T3S function, then bacteria producing these should compete equally well with parental bacteria for survival during co-infection of mice. To facilitate these competition infection experiments, we utilised our prior knowledge that Cm^R^ bacteria containing a polar mutation within the gene encoding for a inner membrane oligo-dipeptide/nickel ABC transporter permease (annotated as YPTB0523 in *Y. pseudotuberculosis* IP32953) successfully competes with parental bacteria for equal colonization of organ tissues in orally infected mice (UA, unpublished). Therefore, we introduced this polar mutation into the orthologous YPK_3687 locus (as annotated in *Y. pseudotuberculosis* YPIII) of our temperature sensitive Δ*yopN, tyeA* mutant as well as all four regulatory competent YopN-TyeA hybrid producing bacteria and the YopN_*YpsYen*_ producing bacteria (that can no longer naturally produce any hybrid). This gave rise to six new strains that now are all Cm^R^ to serve as a convenient selective marker to distinguish them from the Cm^S^ parental bacteria during the process of determining CFU counts derived from spleens dissected on day 4 from groups of five mice orally co-infected with a known input ratio of both parent (Cm^S^) and mutant (Cm^R^) bacteria. As a control, we also co-infected with parental bacteria (Cm^S^) and the isogenic mutant containing only the additional polar mutation introduced into the YPK_3687 gene (Cm^R^). As anticipated from unpublished data, a competitive index (CI) value of 0.9 confirms that this YPK_3687 polar mutation in parental bacteria (*yopN*_wt_), does not compromise the ability of these Cm^R^ bacteria to compete with Cm^S^ parent (also *yopN*_wt_) for systemic spreading and spleen colonization (Figure 8 and Table S2) (UA, unpublished). On the other hand, the Cm^R^ Δ*yopN, tyeA* mutant fared extremely poorly in competition with the Cm^S^ parent containing the wild type *yopN* allele (Figure 8 and Table S2; *P*=0.0079, **). At least in part, the very low CI score of 0.00008 for the Δ*yopN, tyeA* mutant reflects its inability to grow at body temperature. On the other hand, YopN_*YpsYen*_ producing bacteria possessed a CI score of 1.04 (Figure 8 and Table S2; *P*=0.8413). This suggests that while singular YopN and TyeA are being produced, it matters not whether these bacteria also produce the larger hybrid form. Interestingly, the YopN _278(F+1_)TyeA, YopN _278(F+1_)_, SD_TyeA, YopN _287(F+1_)TyeA and YopN _287(F+1_)_, SD_TyeA hybrid producing bacteria presented CI values of 0.096 (*P*=0.0317, *), 0.032 (*P*=0.0079, **), 0.059 (*P*=0.0159, *) and 0.135 (*P*=0.0317, *) respectively, which were all significantly lower than parental control bacteria (Figure 8 and Table S2). Significantly, only two of these hybrid producing bacteria were compromised in polarized secretion (see Figure 6). Hence, these sensitive competitive survival co-infection experiments revealed that all four YopN-TyeA hybrids are not the functional equal of YopN and TyeA produced as independent polypeptides; an observation missed when using *in vitro* based assays that evidently lack the discriminatory sensitivity to resolve subtle biologically relevant imperfections in T3SS activity.

**Figure 9:**
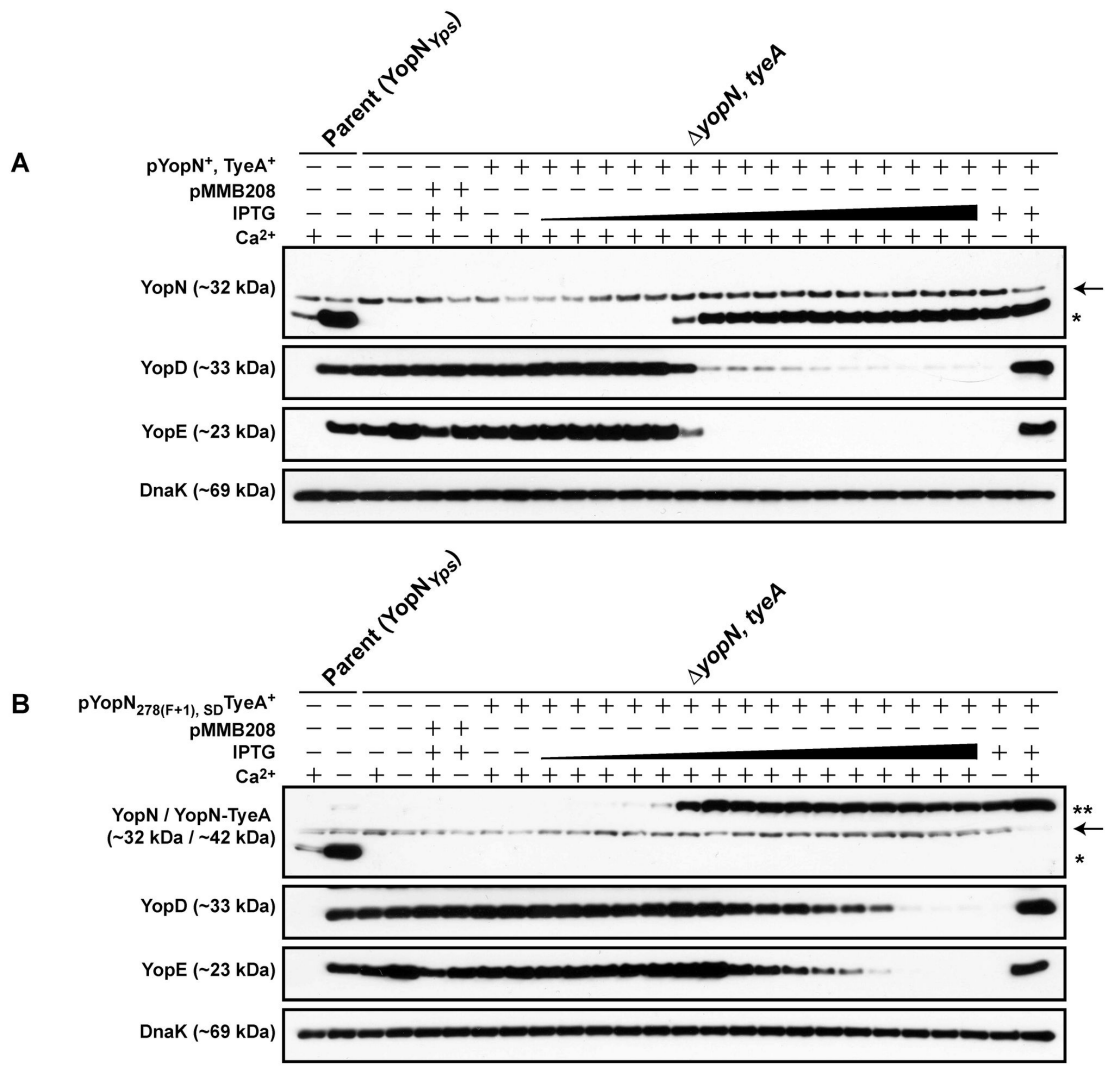
Minimal level of YopN and TyeA required for regaining Yop synthesis control. A deregulated *yopN* and *tyeA* double mutant of *Y*. *pseudotuberculosis* (YPIII/pIB8201a) expressing either an IPTG inducible variant of native YopN and TyeA (A; pYopN^+^, TyeA^+^) or an engineered YopN-TyeA hybrid (B; pYopN _278_(_F+1_)_, SD_TyeA^+^) was grown at 37 °C in T3S restrictive (+Ca^2+^) or T3S permissive (−Ca^2+^) conditions. Protein associated with pelleted bacteria was fractionated by 12% acrylamide SDS–PAGE and detected by Western blot using polyclonal anti-YopN, anti-YopD and anti-YopE antiserum. As a bacterial loading control we probe levels of the cytoplasmic molecular chaperone DnaK with anti-DnaK antibodies. Variable ectopic expression of native YopN and TyeA or the engineered hybrid variant was achieved via the incremental increase in the final concentration of IPTG added to the growth media (0.001 mM, 0.002 mM, 0.003 mM, 0.004 mM, 0.005 mM, 0.01 mM, 0.02, mM, 0.025 mM, 0.03 mM, 0.035 mM, 0.04 mM, 0.045 mM, 0.05 mM, 0.1 mM, 0.2 mM and 0.3 mM respectively). The `−´ symbol indicates the absence of IPTG, while `+´ indicates a final concentration of 0.4 mM. Strains: Parent (YopN_*Yps*_), YPIII/pIB102; Δ*yopN, tyeA* double mutant, YPIII/pIB8201a; The single asterisk (`*´) indicates singular YopN, while the double asterisk (`**´) represents the YopN-TyeA hybrid variant. The arrow (→) is pointing toward a non-specific protein band recognized by the anti-YopN antiserum. Approximate molecular mass values shown in parentheses were deduced from primary amino acid sequences.

We were curious to identify a reason for the slight virulence attenuation of the YopN-TyeA hybrid producing bacteria. The fact that the hybrids YopN _278(F+1_)TyeA and YopN _278(F+1_)_, SD_TyeA displayed a subtle increase in non-polarized Yop secretion (see Figure 6) hinted that the fine-tuning of Yop secretion control is a reason for virulence attenuation. To investigate this, an *in vitro* regulatory assay was designed that had an enhanced discriminatory power over traditional T3S assays. Two IPTG-inducible expression constructs based upon pMMB208 were generated; the first contained native full-length and overlapping *yopN* and *tyeA* alleles (pAA269) and the second with the engineered *yopN*_278(F+1_)_, SD_ *tyeA* allele (pAA271) whose hybrid product caused the most virulence attenuation (see Figure 8 and Table S2). Using the fact that the Δ*yopN, tyeA* double mutant is deregulated for Yop synthesis, even at the non-permissive high Ca^2+^ conditions (see Figure 5), we examined how efficient the two expression constructs were at restoring feedback inhibitory control i.e. preventing Yops synthesis at high Ca^2+^ conditions. We did this by progressively titrating into the growth medium increasingly higher concentrations of IPTG. It was very evident that as soon as ectopic singular YopN (~32 kDa) and TyeA (not shown) expression was detectable (using as little as 0.01 mM IPTG) cessation of YopE and to a lesser extent YopD synthesis occurred concomitantly (Figure 9A). In contrast, although ectopic YopN _278(F+1_)_, SD_TyeA hybrid (~42 kDa) protein was detectable at an even lower IPTG concentration (using as little as 0.04 mM IPTG), complete cessation of YopE synthesis, and to a lesser extent YopD synthesis, required at least a 5-fold higher IPTG concentration than was used for native YopN and TyeA expression (Figure 9B). However, this delay in Yop synthesis inhibition cannot be explained by insufficient accumulation of YopN _278(F+1_)_, SD_TyeA, which was at least the equivalent of maximal levels of singular YopN even at low IPTG doses. Hence, we can only assume that the action of the hybrid in instigating repression – presumably by resetting the YopN secretion plug in the channel – is comparatively sluggish. Thus, we believe hybrid producing mutants are routinely less fit in infected animals because they are unable to respond rapidly to coordinate changes in Ysc-Yop synthesis and secretion in accordance with environmental flux encountered when in the host animal.

### Establishing a Frame-shifting Mechanism for YopN-TyeA Hybrid Production

**Figure 10:**
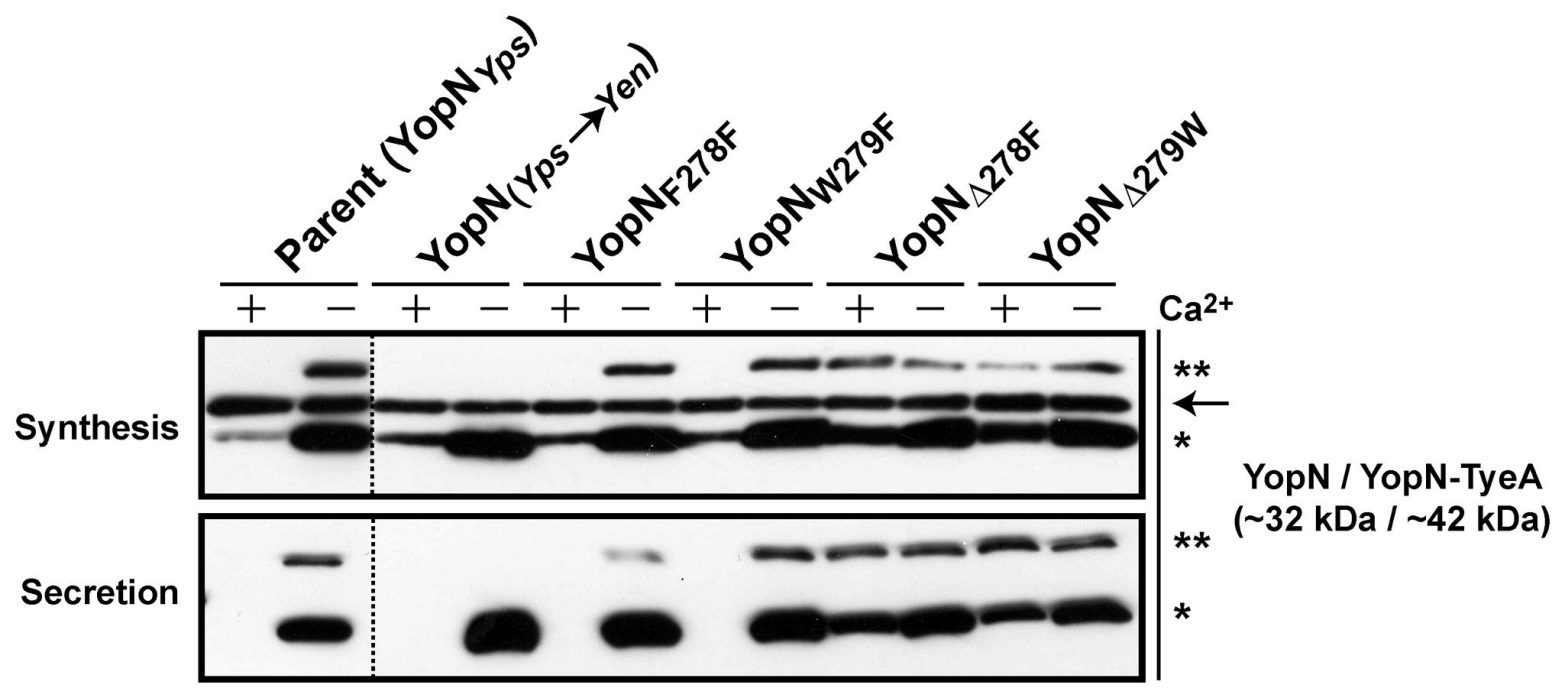
Analysis of Yop synthesis and secretion in YopN mutants manipulated at codons 278 or 279. Overnight cultures of *Y. pseudotuberculosis* were sub-cultured into BHI medium in the presence (+) or absence (-) of calcium ions at 26°C for 1 hour and at 37°C for 3 hours. Protein in the total bacterial suspension (Synthesis) and free in the cleared culture supernatant (Secretion) were collected, fractionated by 12% acrylamide SDS-PAGE, wet-blotted onto PDVF membrane and then detected using rabbit polyclonal anti-YopN. The arrow (→) is pointing toward a non-specific protein band recognized by the anti-YopN antiserum. The single asterisk (*) highlights the single YopN polypeptide, while the double asterisk (**) indicates the larger YopN-TyeA hybrid protein. Lanes: Parent (YopN_*Yps*_), YPIII/pIB102; YopN_*Yps*→*Yen*_, YPIII/pIB8214; YopN_F278F_, YPIII/pIB8215; YopN_W279F_, YPIII/pIB8216; YopN_Δ278F_, YPIII/pIB8217; YopN_Δ279W_, YPIII/pIB8218. Approximate molecular mass values shown in parentheses were deduced from primary amino acid sequences.

The mechanism for formation of the naturally occurring YopN-TyeA hybrid in *Y. pestis* was proposed to be a +1 translational frame-shifting event instigated by a putative ribosomal pausing site ‘UUU-UGG’ encompassing codons F_278_ and W_279_ within the 3´-end of *yopN* mRNA [39]. Given the existence of identical *yopN* sequence in *Y. pseudotuberculosis* and *Y. pestis* (see Figure 2), one might assume for this potential frame-shifting mechanism to be shared between the two species. However, this could not be confirmed by mass spectroscopy because our numerous attempts to determine the protein sequence of native YopN-TyeA were fruitless (data not shown), a situation also experienced by others [39]. Therefore, we proceeded to target the putative ‘UUU-UGG’ ribosomal pausing sequence by site-directed mutagenesis in *Y. pseudotuberculosis*. Four *yopN* mutations were generated; the first a silent F_UUU_ F_UUC_ mutation to give YopN_F278F_, the second a missense W_UGG_ F_UUC_ mutation to give YopN_W279F_, while the remaining two were clean deletions of codon 278 and 279 to give YopN_Δ278F_ and YopN_Δ279W_, respectively. All four mutants were grown in BHI media that was either T3S–restrictive (plus Ca^2+^) or T3S–permissive (minus Ca^2+^). Critically, all four bacteria retained the ability to synthesize and secrete a YopN-TyeA chimeric protein of ~42 kDa (Figure 10). However, it was evident that bacteria producing YopN_Δ278F_ or YopN_Δ279W_ had lost the capacity to maintain control of Ysc-Yop T3S, since product was constitutively made and secreted regardless of Ca^2+^ concentration (Figure 10). Hence, the codons 278 and 279 are relevant for YopN activity, but on their own are not solely responsible for the frame-shifting event. This must mean that the frame-shifting mechanism is more complex than previously appreciated, requiring more than just a ribosomal pausing site. In fact, probably no single feature is alone responsible, with additional architectural features neighbouring the site bound to make necessary contributions.

## Discussion

The InvE family of T3S regulators primarily exist as singular proteins [33]. However, a subset of these present in the Ysc-Yop evolutionary clade of T3SSs exist as two separate polypeptides i.e. in the form of YopN- and TyeA-like proteins [2]. This study revealed that *Y. pseudotuberculosis* YopN and TyeA can be synthesised as a singular YopN-TyeA stable polypeptide, corroborating existence of a naturally occurring YopN-TyeA hybrid first observed in *Y. pestis* [39]. *In cis* mutants of *Y. pseudotuberculosis* engineered to synthesize solely the YopN-TyeA hybrid proved a useful tool to thoroughly probe function. At least *in vitro*, YopN-TyeA hybrids are efficiently secreted and can themselves control the low Ca^2+^-dependent T3S of other Yops. However, shortcomings of this secretion control first revealed during bacteria-target eukaryotic cell contact, impact negatively on their ability to survive during *in vivo* co-infection of mice. Hence, while YopN-TyeA fusions are functional in calcium regulation of Yops secretion, these alleles are not as good as wild type YopN and TyeA produced as discrete polypeptides. As yet, we have no molecular comprehension for why some type III secretion systems prefer two polypeptides and others prefer one. More apparent was that our standard *in vitro* assays routinely used to assess defects in Yop regulatory control can lack the sensitivity to detect subtle abnormalities. It follows though that any such abnormalities identifiable *in vitro*, no matter how subtle, are most likely meaningful in the context of bacterial colonization and survival *in vivo* during an animal model infection.

Although many frame-shifting events are errors in mRNA translation processing that result in mRNA decay and partly completed non-functional products, programmed frame-shifting can be an important translational control mechanism for regulating the production or diversity of protein entities [56-60]. For good reasons, natural YopN-TyeA hybrid production in *Y. pestis* was thought to involve a +1 translational frame-shift brought about by a UUU-UGG ribosomal pausing site at codon positions 278 and 279 of *yopN* mRNA [39]. This raised the possibility of this being a genuine ‘programmed’ frame-shift that had evolved to modulate YopN-TyeA hybrid levels for a physiological purpose. In reality however, disruption of this putative UUU-UGG ribosomal pausing site in *yopN* of *Y. pseudotuberculosis* had no detrimental impact on YopN-TyeA hybrid formation (see Figure 10). This observation could mean that this sequence is not the actual ribosomal pausing site. However, this could not be confirmed as numerous attempts by us and others [39] failed to determine directly the amino acid sequence of purified YopN-TyeA hybrid. On the other hand, it remains feasible that the frame-shifting mechanism utilizes this ribosomal pausing site, but requires influence from architectural features neighbouring the site. As SD sites internal to the open reading frame influence translational pausing [68-70], it is conceivable that composition and location of the putative SD sequence of *tyeA* relative to the upstream pausing site and the downstream *tyeA* initiation codon could affect the extent of YopN-TyeA formation. In addition, frame-shifting can be heavily influenced by the mRNA secondary structure downstream of the pausing site [71,72], and can also be controlled by codon usage and the relative abundance of a given tRNA [73,74]. Thus, a number of epigenetic features have potential to influence frame-shifting events that lead to YopN-TyeA formation.

Epigenetic regulatory elements aside, it is also established that polyamines can enhance +1 frame-shifting [75]. Polyamines are small polycationic molecules ubiquitous in almost all life-forms [76,77]. They have a natural affinity for binding to RNA, which affords them the opportunity to alter protein synthesis in ways that influence multiple cellular functions [76,77]. The prospect that polyamines could influence the frame-shifting event leading to YopN-TyeA hybrid production is tantalising given how they are already linked to controlling T3SS activity in some bacteria [78,79]. Thus, it is apparent that the frame-shifting event leading to YopN-TyeA formation is probably multifaceted; dissecting this mechanism will need to address numerous possible influences.

Engineering a +1 frameshift after codon 278 or codon 287 had the purpose of coercing the production of a YopN-TyeA hybrid. Remarkably, these bulkier hybrids are accumulated to higher levels than the single YopN product, are efficiently secreted and, to varying degrees, also support the T3S control of other Yops. In our minds, the secretion of YopN-TyeA underlines the tolerance that T3SSs display for the secretion of diverse substrates. It seems that the YopN-TyeA polypeptide still boasts a recognisable N-terminal secretion signal [80], can still be piloted for secretion by cognate heterodimeric T3S chaperone composed of YscB and SycN [36,81], and can still unfold itself in a manner that must ensure both controlled and efficient secretion via the Ysc-Yop T3SS [82], and only when the bacteria senses the appropriate environmental signals.

In reality, functional hybrids contain a C-terminal YopN sequence between residues 278 to 286 that barely resembles native YopN, and the residues beyond 287 no longer exist as they are replaced by the TyeA N-terminus (see Figure 2). In particular, a +1 frame-shift after 278 appeared to produce a more defective hybrid – as evidenced by decreased polarized secretion following cell contact and virulence attenuation – then when introducing a +1 frame-shift after codon 287. Consistent with this, the former hybrids contain several more amino acid modifications in the YopN C-terminal sequence. We speculate that these extra changes might be the reason for a more defective hybrid entity. This could be either as a direct consequence of reducing activity of the YopN module or through generating a less flexible TyeA tail module. Since specific deletion of either codon 278 or 279 produced YopN variants with inferior control of Yop synthesis and secretion (see Figure 10), this suggests that the native YopN C-terminal segment has a key role in mediating T3S control. This will be addressed in our future work.

Independent methods have also indicated that at least a portion of YopN can be translocated into eukaryotic cells, in a process that is negatively influenced by the presence of functional TyeA [38,66]. With no known enzymatic activity or intracellular molecular target, the relevance of translocated YopN remains obscure. This study did not attempt to investigate if any of the YopN-TyeA hybrids are translocated into HeLa cell monolayers. If a legitimate role for YopN translocation is identified, the YopN-TyeA hybrids could be used to investigate by what mechanism the presence of a C-terminal TyeA appendage quenches this YopN activity.

As already hinted above, it is also possible that TyeA function within these engineered hybrids is affected. For example, hybrid secretion places this TyeA component outside of the cell, effectively depleting its cytoplasmic pools. Past conjecture has surrounded the secretory status of native TyeA [34,61,62]. However, the current paradigm for Yop secretion control places TyeA in the cytoplasm, functioning as an intracellular anchor facilitating the plugging of the secretory channel by YopN [41]. It is also probable that TyeA fused to the C-terminus of YopN becomes less supple, losing the required flexibility to perform its YopN anchoring role. Structural flexibility of TyeA may be needed for other protein-protein interactions. Aside from YopN, TyeA has been shown to interact with YopD [34,61] and more recently YscG and a hypothetical protein annotated as YPCD1.16C in *Y. pestis* (pYV0009 in *Y. pseudotuberculosis* IP32953) [83]. However, the significance of these reported interactions is unclear; it is not known whether any of them assist TyeA in anchoring the YopN plug to control Yops secretion. Interestingly, the well-studied YopD protein has a role both inside and outside of the bacteria [55,84], theoretically making it available to either non-secreted or secreted TyeA. At any rate, altering the context of TyeA function beyond YopN may explain the reduced fitness in mice (see Figure 8 and Table S2) and the sluggish secretion control (see Figures 6 and 9) of bacteria producing the 278(F+1) series of hybrids. Our YopN-TyeA hybrids, or variants thereof, where cytoplasmic TyeA depletion is forced, could open up unexplored avenues to study the mechanism of TyeA-dependent anchoring of YopN in addition to revealing the biological consequences of these other TyeA-dependent protein-protein interactions.

Another point is that free TyeA produced as an independent polypeptide may affect YopN-TyeA hybrid function. For example, this native TyeA could engage with the YopN component of the hybrid, potentially contributing to the small phenotypic differences we have observed in this study. For this reason we generated two extra hybrids (i.e. the SD-minus YopN _278(F+1_)_, SD_TyeA and YopN _287_(_F+1_)_, SD_TyeA mutants) having a ‘scrambled’ sequence aimed at disrupting a probable SD site of *tyeA* to limit its production. However, western blotting confirmed that all four hybrids (i.e. regardless of the SD sequence being intact or disrupted) produced very little to no detectable free TyeA. First of all, these data cannot substantiate whether the nucleotides ‘AGAGGG’ (see Figure 2) do actually represent a bona fide SD of *tyeA*. In addition, we found no correlation between native free TyeA production and the modest phenotypic defects displayed by the YopN-TyeA chimeras. Therefore, it seems unlikely that a free native TyeA bias (i.e. in the SD^+^ mutants of YopN_278(F+1),_ TyeA and YopN_287(F+1),_ TyeA) can account for the defects in functionality of the hybrids.

Analysis of the InvE family of proteins is adding credence to the concept of a secretion hierarchy among the middle translocator substrates and the late effector secretion substrates [19,25-31]. This is attractive because it fits nicely with the original tenet that the translocon pore should form in the host cell plasma membrane before substrates destined for translocation through this pore are actually secreted. As described in a recent review [41], there is some evidence that *Yersinia* preferentially secretes YopB and YopD translocator substrates in Ca^2+^ rich media that otherwise prevents Yop effector secretion. In these situations (i.e. prior to cell contact or in the presence of elevated calcium), it is the YopN/SycN/YscB/TyeA complex that inhibits effector secretion in order to prioritise translocator secretion [38,66]. This cannot be true of all situations however, since unstimulated *Y. pseudotuberculosis* contains on their surface both Yop translocators and Yop effectors in equal measure that proceed to form translocation-competent Yop complexes [63]. Moreover, our western blotting experiments probing for levels of both the YopD translocator and the YopE effector in defined *yopN-tyeA* mutants did not reveal any preferential secretion of YopD in Ca^2+^ replete conditions *in vitro* (e.g. see Figure 5). Hence, if hierarchical secretion does exist in *Y. pseudotuberculosis*, YopN and TyeA apparently do not orchestrate it. Interestingly, it appeared that basal levels of YopD are always subtly higher than YopE both *in vitro* (see Figure 9) and during cell contact (see Figure 6). This is reminiscent of recent studies purporting to a hierarchical Yops expression profile instigated through the translation-inhibitory effects of YopD/LcrH complexes differentially bound to the various 5´-untranslated regions of *yop* mRNAs [84,85]. Arguably therefore, any preference for prioritising translocator secretion may have its genesis in regulating cytoplasmic pools of pre-made Yops.

In summary, this study was unable to show any direct support for YopN and TyeA in orchestrating hierarchical secretion among the Yop translocator and effector substrates. However, it was clear that no matter how subtle the regulatory defects were in controlling Yop synthesis and secretion *in vitro*, they had a direct impact on the *in vivo* fitness of *Yersinia* bacteria. Our outcomes also lead us to posit that work aimed at further unravelling the specific mechanisms of feedback inhibition and post-transcriptional control of *yops* expression through YopD function could have fertile consequences for understanding how *Yersinia* might consider prioritising different Yop substrates for secretion.

## Supporting Information

Figure S1 **Analysis of free TyeA synthesis and secretion in synthetic YopN-TyeA chimeric mutants.** Overnight cultures of *Y. pseudotuberculosis* were sub-cultured into BHI medium in the absence of calcium ions at 26°C for 1 hour and at 37°C for 3 hours. At the time of temperature up-shift, 0.4 mM IPTG was added to all cultures. Protein in the total bacterial suspension (Synthesis) and free in the cleared culture supernatant (Secretion) were collected, fractionated by 15% acrylamide SDS-PAGE, wet-blotted onto PDVF membrane and then detected using rabbit polyclonal anti-TyeA antibodies. The arrow (→) point towards a non-specific protein band recognized by the anti-TyeA antiserum. The single asterisk (*) highlights the larger YopN-TyeA hybrid protein. Lanes are *Y. pseudotuberculosis* Δ*yopN, tyeA* (YPIII/pIB8201a) also containing pYopN, TyeA^+^ (pAA304), empty vector (pMMB208), pYopN_278(F+1)_, TyeA^+^ (pAA306), pYopN_278(F+1), SD_, TyeA^+^ (pAA307), pYopN_287(F+1)_, TyeA^+^ (pAA308), or pYopN_287(F+1), SD_, TyeA^+^ (pAA309). Approximate molecular mass values shown in parentheses were deduced from primary amino acid sequences.
(TIF)

Figure S2 **YopN-TyeA hybrid-producing bacteria spawn external YscF multimers.** *Yersinia* strains were grown in non permissive T3S media (plus Ca^2+^). Where indicated (+), the membrane-impermeable chemical cross-linker BS^3^ was added to the bacteria. After being quenched with Tris-HCl, bacteria pellets were solubilized in sample buffer and then protein fractionated by 12% acrylamide SDS-PAGE. After wet-transfer to PVDF, YscF was detected with immune-absorbed monospecific anti-YscF antiserum. Non-cross-linked monomeric YscF was observed in all lanes except the Δ*yscF* null mutant control. Cell-surface YscF multimers were observed in all lanes except for the Δ*yscF* null mutant control as well as the YscF^+^, but T3SS-defective, Δ*yscU, lcrQ* null mutant control. The predicted molecular mass of monomeric YscF is given in parenthesis, while approximate sizes of protein molecular weight standards are given to the right. Strains: Parent (YopN_*Yps*_), YPIII/pIB102; Δ*yopN* null mutant, YPIII/pIB82; Δ*tyeA* null mutant, YPIII/pIB801a; Δ*yopN, tyeA* double mutant, YPIII/pIB8201a; YopN _278(F+1)_TyeA, YPIII/pIB8205; YopN _278(F+1), SD_TyeA, YPIII/pIB8206; YopN _287(F+1)_TyeA, YPIII/pIB8210; YopN _287(F+1), SD_TyeA, YPIII/pIB8211; Δ*yscF* null mutant, YPIII/pIB202; Δ*yscU, lcrQ* double mutant, YPIII/pIB75-26.
(TIF)

Figure S3 **Low calcium response growth phenotypes of *Y. pseudotuberculosis* producing YopN-TyeA hybrids.** Bacteria were grown at 37°C in TMH medium supplemented with 2.5 mM CaCl_2_ (plus Ca^2+^; **A**) or non-supplemented (minus Ca^2+^; **B**). Two different growth phenotypes were detected: TS – bacteria are sensitive to elevated temperature regardless of the presence or absence of calcium (Δ*yopN* and/or Δ*tyeA* null mutants) and, CD – calcium dependent growth (all remaining strains). Strains: Parent (YopN_*Yps*_), YPIII/pIB102; Δ*yopN* null mutant, YPIII/pIB82; Δ*tyeA* null mutant, YPIII/pIB801a; Δ*yopN, tyeA* double mutant, YPIII/pIB8201a; YopN _278(F+1)_TyeA, YPIII/pIB8205; YopN _278(F+1), SD_TyeA, YPIII/pIB8206; YopN _287(F+1)_TyeA, YPIII/pIB8210; YopN _287(F+1), SD_TyeA, YPIII/pIB8211.
(TIF)

Table S1 **Oligonucleotides used in this study.**
(PDF)

Table S2 **Competitive index for mice colonization.**
(PDF)
