# Supplementary figures and images for: Genetically Engineered Frameshifted YopN-TyeA Chimeras Influence Type III Secretion System Function in Yersinia pseudotuberculosis
Source: PLoS One. 2013 Oct 3;8(10):e77767. doi: 10.1371/journal.pone.0077767 (PMC3789692; doi:10.1371/journal.pone.0077767)

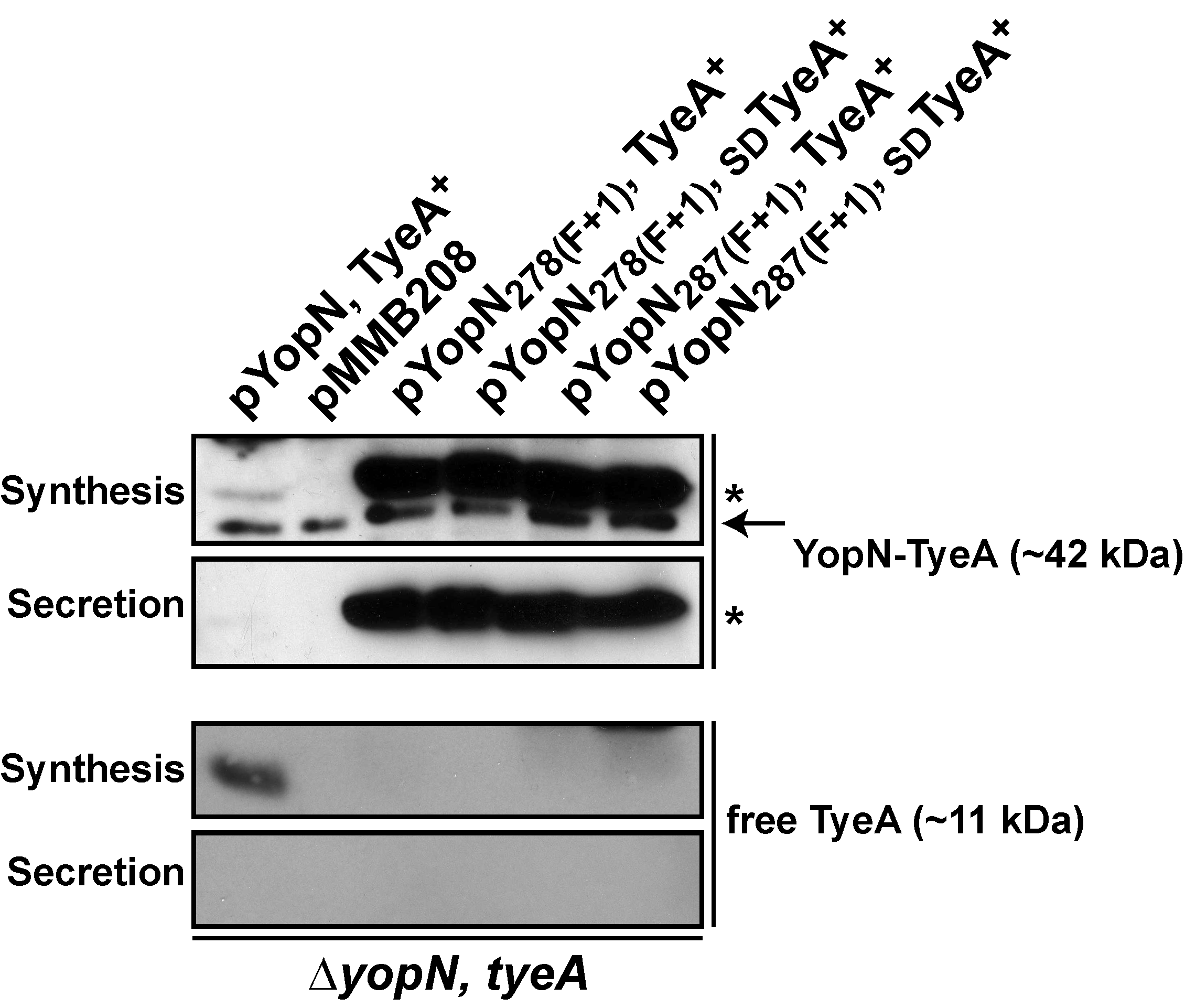

Supplement: Figure S1 — Analysis of free TyeA synthesis and secretion in synthetic YopN-TyeA chimeric mutants. Overnight cultures of Y. pseudotuberculosis were sub-cultured into BHI medium in the absence of calcium ions at 26°C for 1 hour and at 37°C for 3 hours. At the time of temperature up-shift, 0.4 mM IPTG was added to all cultures. Protein in the total bacterial suspension (Synthesis) and free in the cleared culture supernatant (Secretion) were collected, fractionated by 15% acrylamide SDS-PAGE, wet-blotted onto PDVF membrane and then detected using rabbit polyclonal anti-TyeA antibodies. The arrow (→) point towards a non-specific protein band recognized by the anti-TyeA antiserum. The single asterisk (*) highlights the larger YopN-TyeA hybrid protein. Lanes are Y. pseudotuberculosis ΔyopN, tyeA (YPIII/pIB8201a) also containing pYopN, TyeA+ (pAA304), empty vector (pMMB208), pYopN278(F+1), TyeA+ (pAA306), pYopN278(F+1), SD, TyeA+ (pAA307), pYopN287(F+1), TyeA+ (pAA308), or pYopN287(F+1), SD, TyeA+ (pAA309). Approximate molecular mass values shown in parentheses were deduced from primary amino acid sequences. (TIF) [file pone.0077767.s001.tif]

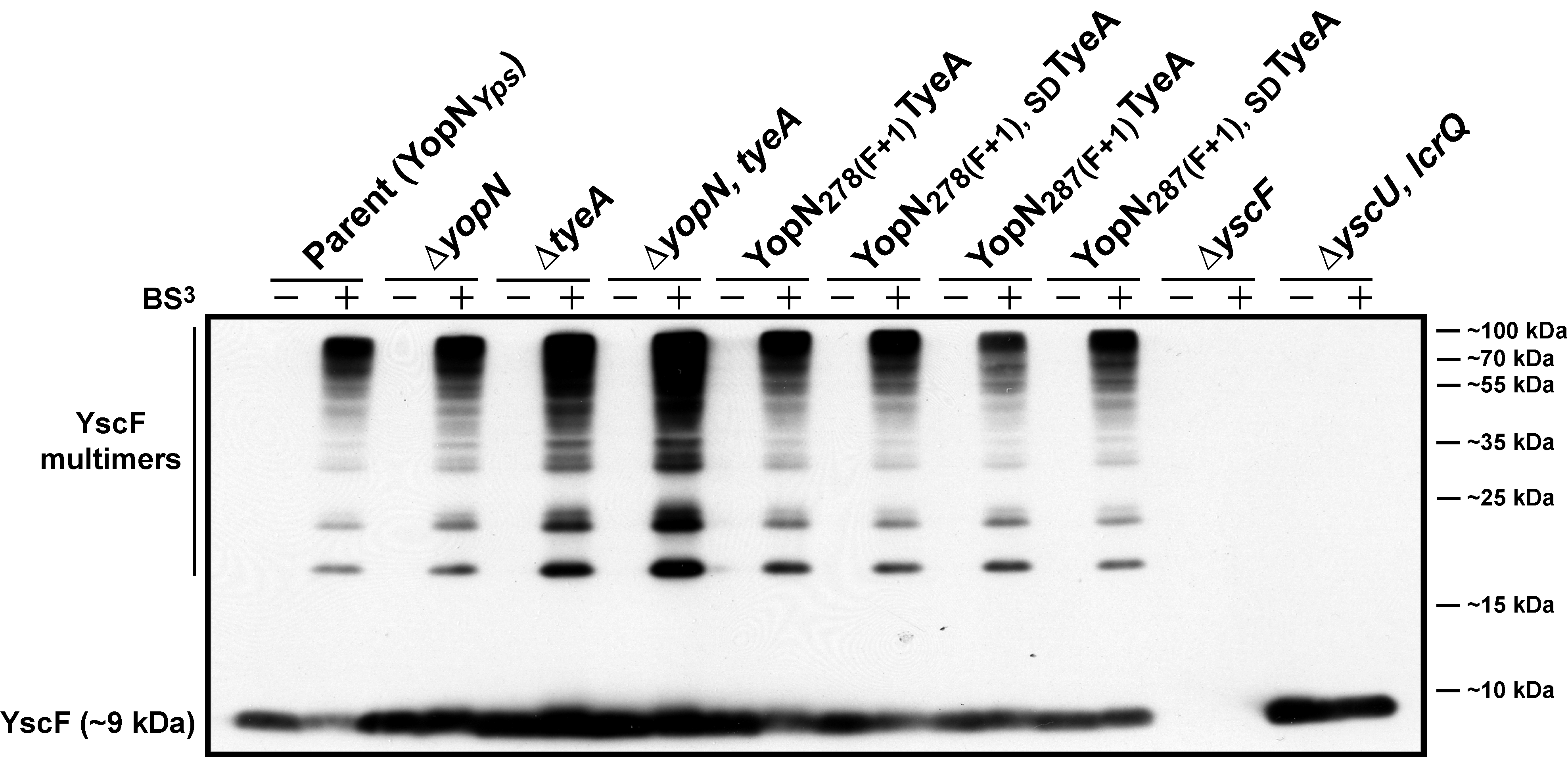

Supplement: Figure S2 — YopN-TyeA hybrid-producing bacteria spawn external YscF multimers. Yersinia strains were grown in non permissive T3S media (plus Ca2+). Where indicated (+), the membrane-impermeable chemical cross-linker BS3 was added to the bacteria. After being quenched with Tris-HCl, bacteria pellets were solubilized in sample buffer and then protein fractionated by 12% acrylamide SDS-PAGE. After wet-transfer to PVDF, YscF was detected with immune-absorbed monospecific anti-YscF antiserum. Non-cross-linked monomeric YscF was observed in all lanes except the ΔyscF null mutant control. Cell-surface YscF multimers were observed in all lanes except for the ΔyscF null mutant control as well as the YscF+, but T3SS-defective, ΔyscU, lcrQ null mutant control. The predicted molecular mass of monomeric YscF is given in parenthesis, while approximate sizes of protein molecular weight standards are given to the right. Strains: Parent (YopNYps), YPIII/pIB102; ΔyopN null mutant, YPIII/pIB82; ΔtyeA null mutant, YPIII/pIB801a; ΔyopN, tyeA double mutant, YPIII/pIB8201a; YopN 278(F+1)TyeA, YPIII/pIB8205; YopN 278(F+1), SDTyeA, YPIII/pIB8206; YopN 287(F+1)TyeA, YPIII/pIB8210; YopN 287(F+1), SDTyeA, YPIII/pIB8211; ΔyscF null mutant, YPIII/pIB202; ΔyscU, lcrQ double mutant, YPIII/pIB75-26. (TIF) [file pone.0077767.s002.tif]

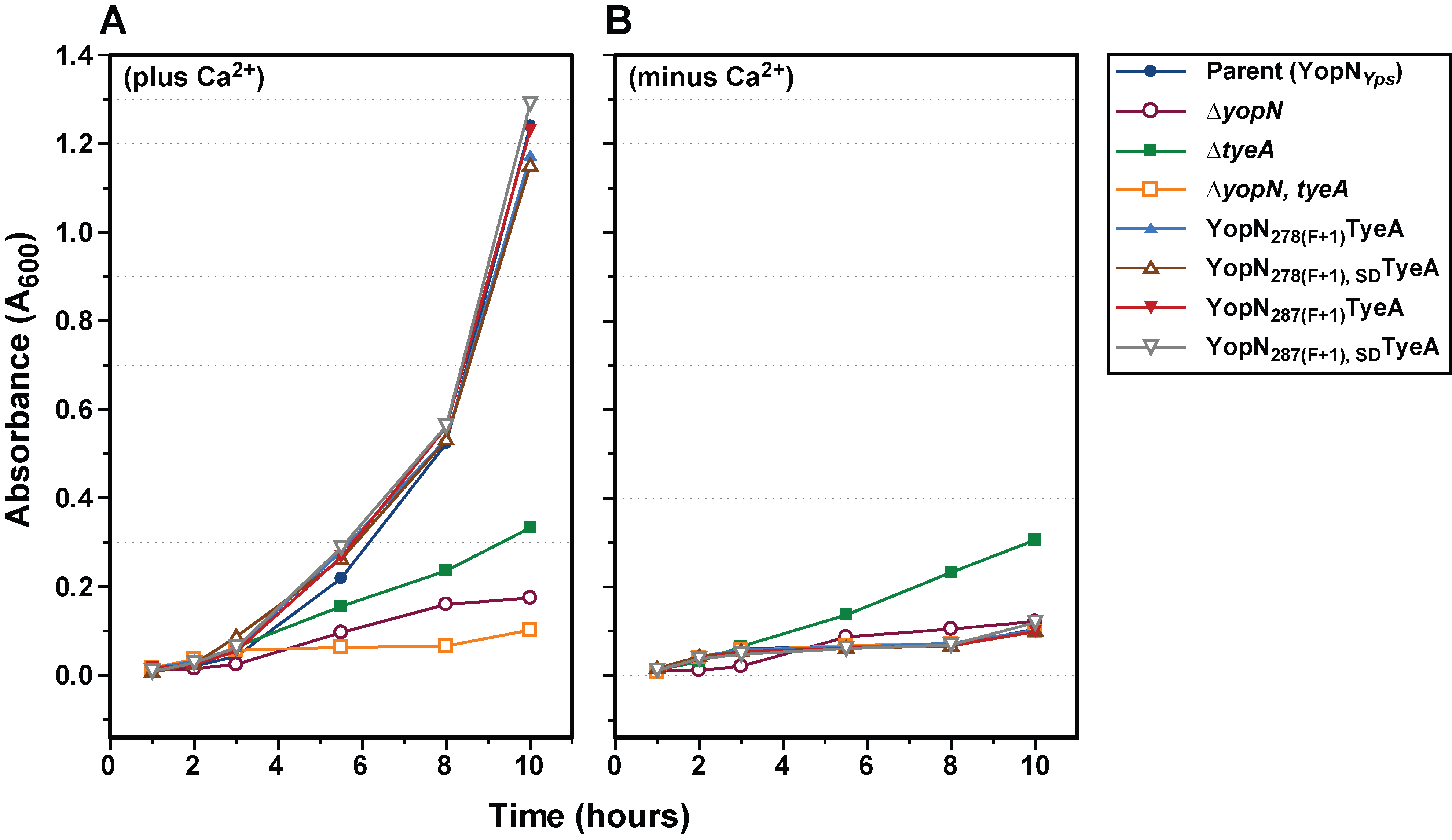

Supplement: Figure S3 — Low calcium response growth phenotypes of Y. pseudotuberculosis producing YopN-TyeA hybrids. Bacteria were grown at 37°C in TMH medium supplemented with 2.5 mM CaCl2 (plus Ca2+; A) or non-supplemented (minus Ca2+; B). Two different growth phenotypes were detected: TS – bacteria are sensitive to elevated temperature regardless of the presence or absence of calcium (ΔyopN and/or ΔtyeA null mutants) and, CD – calcium dependent growth (all remaining strains). Strains: Parent (YopNYps), YPIII/pIB102; ΔyopN null mutant, YPIII/pIB82; ΔtyeA null mutant, YPIII/pIB801a; ΔyopN, tyeA double mutant, YPIII/pIB8201a; YopN 278(F+1)TyeA, YPIII/pIB8205; YopN 278(F+1), SDTyeA, YPIII/pIB8206; YopN 287(F+1)TyeA, YPIII/pIB8210; YopN 287(F+1), SDTyeA, YPIII/pIB8211. (TIF) [file pone.0077767.s003.tif]
