# Supplementary material for: Genetically Engineered Frameshifted YopN-TyeA Chimeras Influence Type III Secretion System Function in Yersinia pseudotuberculosis
Source: PLoS One. 2013 Oct 3;8(10):e77767. doi: 10.1371/journal.pone.0077767 (PMC3789692; doi:10.1371/journal.pone.0077767)
Supplement: Table S1 — Oligonucleotides used in this study. (PDF) [file pone.0077767.s004.pdf]

Supplementary Table S1. Oligonucleotides used in this study

Purpose/vector	Oligonucleotide name and sequence
Mutagenesis	
pAA256 (*tyeA*$_{\Delta 19-59}$)	ptyeAd20-59a, 5´-AAG G*CT CGA G*GG ATA TAG ACT CGG TGA-3´ (*Xho*I) and ptyeAd20-59b, 5´-GCG CTT GTC AAC CAG TGC-3´
	ptyeAd20-59c, 5´-GCA CTG GTT GAC AAG CGC TTT AGC GAT GAG GAG CAA C-3´ and ptyeAd20-59d, 5´-AAG G*TC TAG A*GC GCT AAC CAC AAT GTC A-3´ (*Xba*I)
pSF019 (Δ*yopN*, *tyeA*)	pyopNa1, 5´-AAA *GTC GAC* ACC TAC AAT GCC ATG ACG-3´ (*Sal*I) and pyopNtyeAb, 5´-AGA TAG GTT ATG AAG CGT CG-3´
	pyopNtyeAc, 5´-ACG CTT CAT AAC CTA TCT TTT AGC GAT GAG GAG CAA CGG-3´ and ptyeAd, 5´-AAA *TCT AGA* GAA CGC GCT AAC CAC-3´ (*Xba*I)
pAA251 (*yopN*$_{Yps \rightarrow Yen}$)	pyopNa2, 5´-AAA *GTC GAC* CTG CGG CTT GCG TGA TG-3´ (*Sal*I) and pyopN-23b, 5´-TAT ACC CTC TGA AAA AAA TTG CC-3´
	pyopN-23c, 5´-TTT CAG AGG GTA TAA CTA ATG GCG T-3´ and ptyeAd (*Xba*I)
pAA242 (*yopN*$_{278(F+1)}$*tyeA*)	pyopNa2 (*Sal*I) and pyopN278+1b, 5´-CCA AAT CCT TTA ACT TGG TCA-3´
	pyopN278+1c, 5´-CCA AGT TAA AGG ATT TGG CAA TTT TTT TCA GA-3´ and ptyeAd (*Xba*I)
pAA243 (*yopN*$_{278(F+1),\ SD}$*tyeA*)	pyopNa2 (*Sal*I) and ptyeA-SDb, 5´-ACG TTG AAA AAA ATT GCC AAA TCC TTT AAC TTG GTC-3´
	ptyeA-SDc, 5´-GGA TTT GGC AAT TTT TTT CAA CGT GTA AAA CTA ATG GCG T-3´ and ptyeAd (*Xba*I)
pAA247 (*yopN*$_{287(F+1)}$*tyeA*)	pyopNa2 (*Sal*I) and pyopN287+1b, 5´-ACG CCA TAG TTT TAC CCT CTG-3´
	pyopN287+1c, 5´-CAG AGG GTA AAA CTA TGG CGT ACG ACC TTT CTG-3´ and ptyeAd (*Xba*I)
pAA248 (*yopN*$_{287(F+1),\ SD}$*tyeA*)	pyopNa2 (*Sal*I) and ptyeA-SDb2, 5´-CCA TAG TTT TGC CTT CGG AAA AAA ATT GCC AAA ATC C-3´
	ptyeA-SDc2, 5´-CCG AAG GCA AAA CTA TGG CGT ACG ACC TTT CTG-3´ and ptyeAd (*Xba*I)
pAA252 (*yopN*$_{F278F}$)	pyopNa2 (*Sal*I) and pyopN-24b, 5´-GAA TCC TTT AAC TTG GTC AC-3´
	pyopN-24c, 5´-ATT CTG GCA ATT TTT TTC AGA GG-3´ and ptyeAd (*Xba*I)
pAA253 (*yopN*$_{W279F}$)	pyopNa2 (*Sal*I) and pyopN-26b, 5´-GAA AAA TCC TTT AAC TTG GTC-3´
	pyopN-26c, 5´-GAT TTT TCC AAT TTT TTT CAG AGG-3´ and ptyeAd (*Xba*I)
pAA254 (*yopN*$_{\Delta 278F}$)	pyopNa2 (*Sal*I) and pyopN-28b, 5´-TCC TTT AAC TTG GTC ACT C-3´
	pyopN-28c, 5´-GTT AAA GGA TGG CAA TTT TTT TCA G-3´ and ptyeAd (*Xba*I)

pAA255 (*yopN*$_{\Delta 279W}$)	pyopNa2 (*Sal*I) and pyopN-28b<br>pyopN-29c, 5´-AAG GAT TTC AAT TTT TTT CAG AGG-3´ and ptyeAd (*Xba*I)
pUA066 (YPTB0523 polar mutation)	pFpNQ066, 5´-TAT CGA TAC CGT CGA CTA TTC ACC CTG GCC CTA ACG C-3´ (*Sal*I) and pRpNQ066, 5`-ATG CGG TAC CTC TAG AAG TGC ACC GCC CAC GAG CAG T-`3 (*Xba*I)
Ectopic expression	
pAA269 (pYopN^{+}, TyeA^{+}) and pAA271 (pYopN$_{278(F+1), SD}$TyeA^{+})	pYopN-bla_for, 5´-ACGT*GGATCC*TCTCAGGGAGTAGTTATGACG-3´ (*Bam*HI) and YopN-TyeA_rev, 5´-ACGT*GAATTC*AGATCCTGGCAGAAATGG-3´ (*Eco*RI)
pAA304 (pYopN^{+}, TyeA-Flag®$^{+}$), pAA305 (pYopN$_{Yps \rightarrow Yen}$$^{+}$, TyeA-Flag®$^{+}$), pAA306 (pYopN$_{278(F+1)}$TyeA-Flag®$^{+}$), pAA307 (pYopN$_{278(F+1), SD}$TyeA-Flag®$^{+}$), pAA308 (pYopN$_{287(F+1)}$TyeA-Flag®$^{+}$), pAA309 (pYopN$_{287(F+1), SD}$TyeA-Flag®$^{+}$)	pYopN-bla_for (*Bam*HI) and pYopN-TyeAflag_rev, 5´-ACGT*GAATTC*TCACTTGTCATCGTCGTCCTTGTAGTCATCCAACTCACTCAATTCTTCC-3´ (*Eco*RI)
