# Supplementary material for: Genetically Engineered Frameshifted YopN-TyeA Chimeras Influence Type III Secretion System Function in Yersinia pseudotuberculosis
Source: PLoS One. 2013 Oct 3;8(10):e77767. doi: 10.1371/journal.pone.0077767 (PMC3789692; doi:10.1371/journal.pone.0077767)
Supplement: Table S2 — Competitive index for mice colonization. (PDF) [file pone.0077767.s005.pdf]

Supplementary Table S2. Competitive index for mice colonization

Strain (Cm^R)[a]	Input CFU/mouse[b]	Output CFU/ml[c]	Competitive index[d]	Fold difference[e]
Parental ($YopN_{wt}$)	3.49×10^8 (59.6%)	1) 1.65×10^6 (88.5%)<br>2) 5.97×10^5 (22.9%)<br>3) 4.12×10^6 (9.7%)<br>4) 9.43×10^5 (85.0%)<br>5) 1.24×10^5 (62.6%)	0.9 ± 0.6	na
Δ*yopN*, *tyeA*	2.34×10^8 (39.6%)	1) 0<br>2) 1.0×10^1 (0.006%)<br>3) 0<br>4) 0<br>5) 8.0×10^1 (0.012%)	0.00008 ± 0.00013<br>(**, *P*=0.0079)	11250
$YopN_{Yps \rightarrow Yen}$	3.35×10^8 (49.0%)	1) 5.60×10^7 (97.2%)<br>2) 3.30×10^5 (106.6%)<br>3) 1.06×10^4 (14.8%)<br>4) 5.92×10^4 (21.6%)<br>5) 1.05×10^6 (15.7%)	1.04 ± 0.95<br>(ns, *P*=0.8413)	0.87
$YopN_{278(F+1)}$TyeA	2.56×10^8 (42.6%)	1) 9.72×10^4 (20.6%)<br>2) 1.31×10^3 (1.0%)<br>3) 3.5×10^1 (3.7%)<br>4) 8.22×10^2 (1.7%)<br>5) 7.0×10^1 (0.0003%)	0.13 ± 0.2<br>(*, *P*=0.0317)	6.92
$YopN_{278(F+1), SD}$TyeA	2.06×10^8 (60.3%)	1) 1.39×10^4 (5.0%)<br>2) 1.67×10^3 (0.1%)<br>3) 2.93×10^2 (0.5%)<br>4) 0<br>5) 1.26×10^4 (7.7%)	0.043 ± 0.057<br>(**, *P*=0.0079)	20.93
$YopN_{287(F+1)}$TyeA	2.53×10^8 (45.7%)	1) 0<br>2) 9.4×10^2 (6.9%)<br>3) 5.91×10^5 (1.4%)<br>4) 0<br>5) 8.52×10^2 (1.7%)	0.044 ± 0.062<br>(**, *P*=0.0079)	20.45
$YopN_{287(F+1), SD}$TyeA	7.15×10^8 (54.5%)	1) 6.03×10^4 (29.2%)<br>2) 8.22×10^3 (6.4%)<br>3) 1.29×10^3 (0.5%)<br>4) 0<br>5) 3.2×10^2 (0.2%)	0.135 ± 0.229<br>(*, *P*=0.0317)	6.67

[a] All mutant strains were selected for on the basis of a Cm^R marker incorporated via a polar mutation introduced *in cis* in the chromosome locus YPK_3687 through a single cross-in of the mutagenesis plasmid pUA066.

[b] Calculated from the amount of water consumed per cage and then averaged over 5 mice. In parentheses are percentages that correspond to the proportion of total bacteria (*i.e.*: Cm^S + Cm^R) in the inoculation water that are Cm^R.

[c] Cm^R bacteria recovered from each individual spleen. In parentheses are percentages that correspond to the proportion of total bacteria (*i.e.*: Cm^S + Cm^R) recovered from an individual spleen that are Cm^R.

[d] Competitive index (CI) is a ratio of the proportion of Cm^R CFU recovered from infected mice (expressed as a percentage of total CFU output – *see* footnote ‘c’, Table S2) and the proportion of Cm^R CFU inoculated into mice (expressed as a percentage of total CFU input – *see* footnote ‘b’) when in direct competition with the Cm^S parental strain that is wild type for the *yopN* allele. A value of 1.0 therefore indicates that both parent and mutant bacteria compete equally well in infecting, colonizing and spreading systemically in orally infected mice. On the other hand, values progressively falling below 1.0 are indicative of the degree of attenuation shown by the Cm^R mutant bacteria relative to the parent ($yopN_{wt}$). Numbers are the mean ± standard deviation of independent CI values derived from five individual mice inoculated through contaminated drinking water. Data sets were analyzed using the non-parametric two-tailed Mann-Whitney *U*-test. Analysis was performed using GraphPad Prism version 5.00 for Windows. Differences between mutants and parent with a p-value < 0.05 were considered significant (* and **). ns – no significant difference

[e] Fold difference is the ratio between CI of parental ($yopN_{wt}$) (numerator) and mutated derivatives of *yopN* and *tyeA* (denominator).
